# Supplementary material for: Effect of Polymer Composition on the Optical Properties of a New Aggregation-Induced Emission Fluorophore: A Combined Experimental and Computational Approach
Source: Polymers (Basel). 2023 Aug 24;15(17):3530. doi: 10.3390/polym15173530 (PMC10489943; doi:10.3390/polym15173530)
Supplement: Supplementary file 1 [file polymers-15-03530-s001.zip › polymers-2563586-supplementary.pdf]

Supporting information of the manuscript entitled: “Effect of the polymer composition on the optical properties of a new aggregation-induced emission fluorophore: a combined experimental and computational approach”

Alberto Picchi[1,†], Qinfan Wang[2,†], Cosimo Micheletti[1], Francesco Ventura[1], Jesse Heijkoop[3], Francesco Picchioni[3], Ilaria Ciofini[2], Carlo Adamo[2,*], Andrea Pucci[1,*]

[1] *Dipartimento di Chimica e Chimica Industriale, Università di Pisa, Via Moruzzi 13, 56124 Pisa, Italy;* alberto.picchi@phd.unipi.it (A.P.); cosimo.micheletti@phd.unipi.it (C.M.).
[2] *École nationale supérieure de chimie de Paris and PSL Research University, CNRS, Institute of Chemistry for Life and Health Sciences (i-CLeHS), FRE2027, 11, rue Pierre et Marie Curie, F-75005 Paris, France;* ilaria.ciofini@chimieparistech.psl.eu *(I.C.);* carlo.adamo@chimieparistech.psl.eu (C.A.).
[3] *Department of Chemical Engineering, Product Technology, University of Groningen, 9747 AG Groningen, The Netherlands;* f.picchioni@rug.nl (F.P.)

* Correspondence: andrea.pucci@unipi.it (A.P.); carlo.adamo@chimieparistech.psl.eu (C.A.).

† *These authors equally contributed*

1. Experimental details 2
2. Photos 21
3. Computational details 22
4. References 30

1. Experimental details

UV-vis spectroscopy

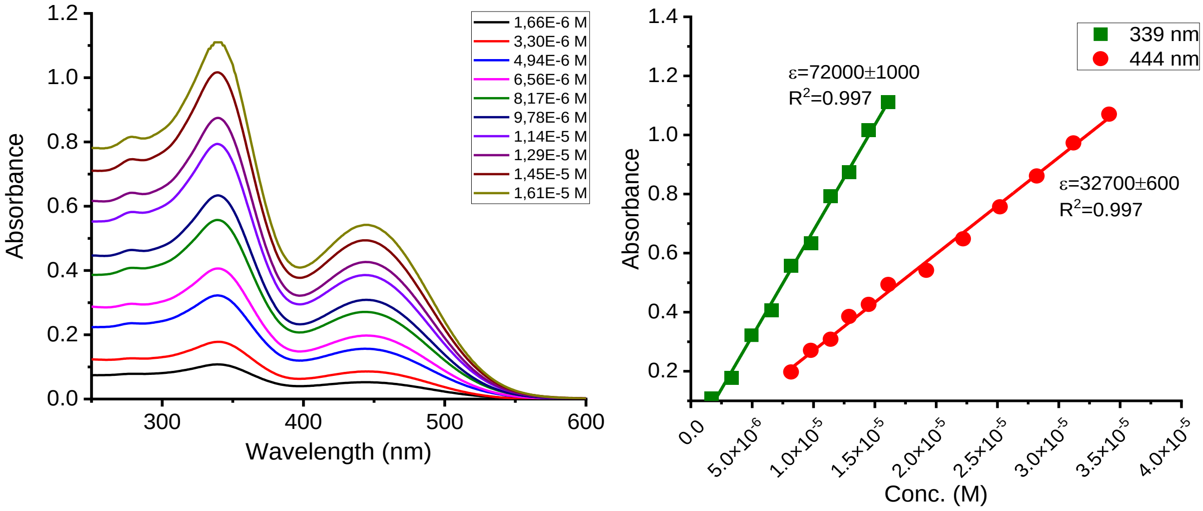

Figure S1: a) UV-vis absorption spectra of TPE/BPAN in THF at different concentrations; b) Maximum absorbance vs. concentration for the two peaks observed in the absorption spectra (ε = molar extinction coefficient).

DLS analysis

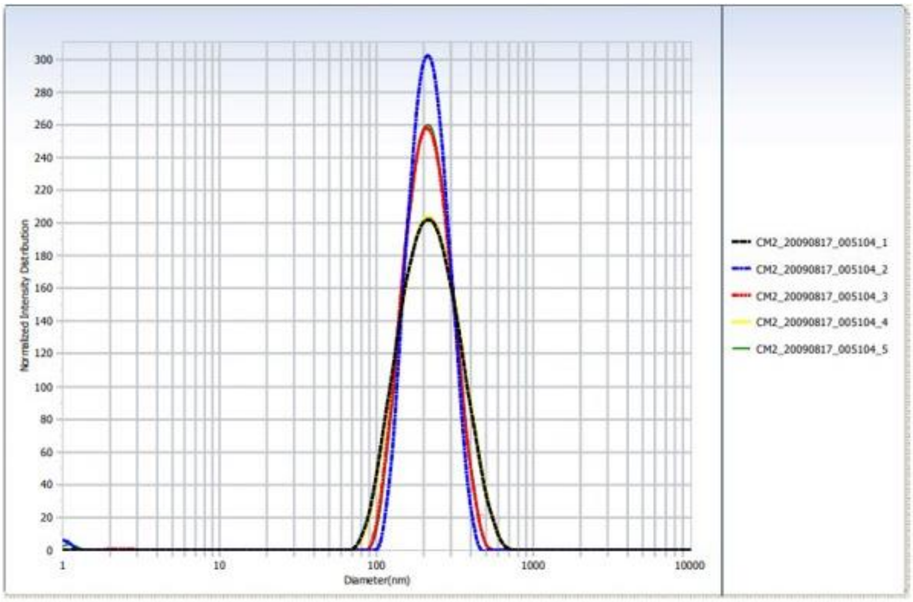

No	Data	Repet. No	pH	Ave. Diameter(nm)	Polydispersity Index	D (10%) (nm)	D (50%) (nm)	D (90%) (nm)
1	CM2_20090817_005104_1	1	NA	202.9	0.114	119.3	206.2	361.0
2	CM2_20090817_005104_2	2	NA	208.5	0.049	140.9	203.7	295.5
3	CM2_20090817_005104_3	3	NA	204.0	0.061	130.9	201.4	312.0
4	CM2_20090817_005104_4	4	NA	205.7	0.101	120.8	207.9	362.4
5	CM2_20090817_005104_5	5	NA	207.0	0.057	132.4	203.5	314.0

Figure S2: DLS size distribution of TPE-BPAN aggregates in a 10/90 vol.% THF/H_2O mixture.

TGA

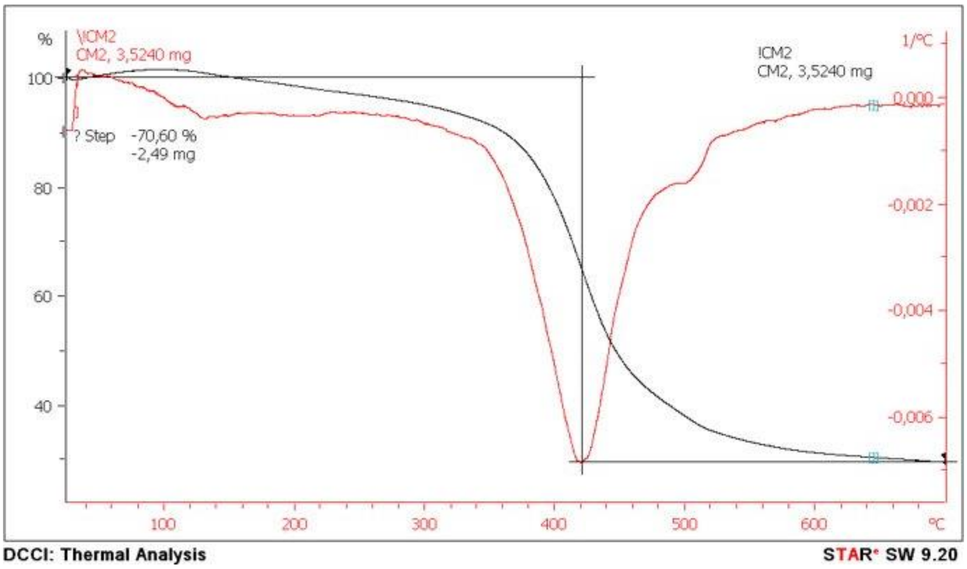

Figure S3: Thermogravimetric analysis of TPE-BPAN.

XRD

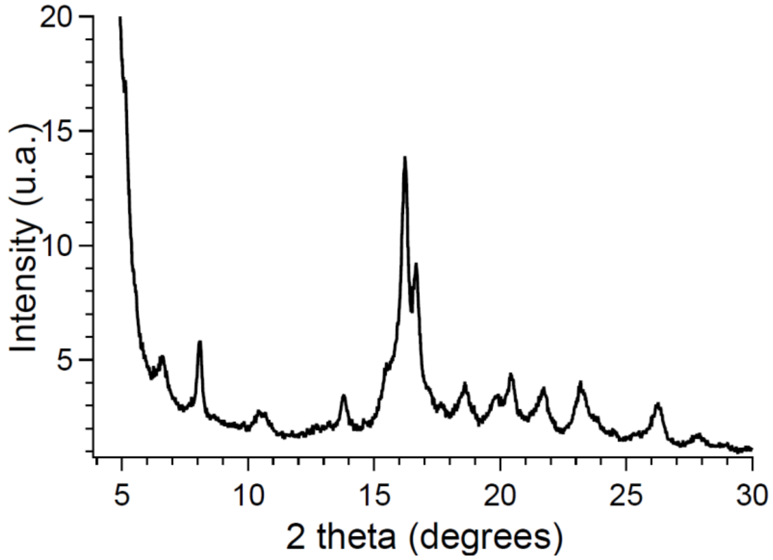

Figure S4: XRD pattern of TPE-BPAN.

NMR spectra

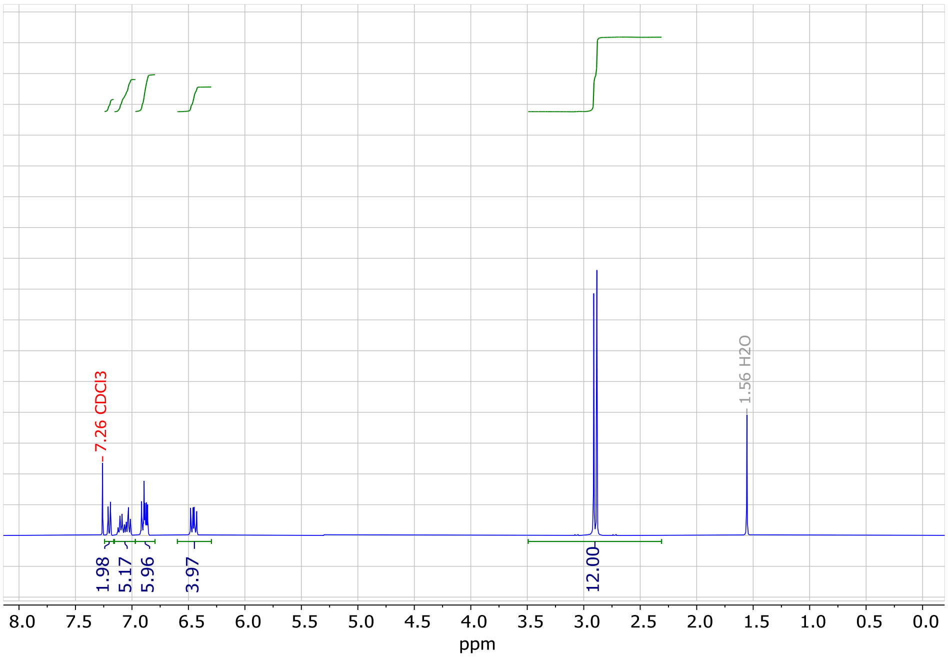

Figure S5: ^{1}H NMR spectrum (400 MHz, $CDCl_3$, 25°C) of TPE-Br.

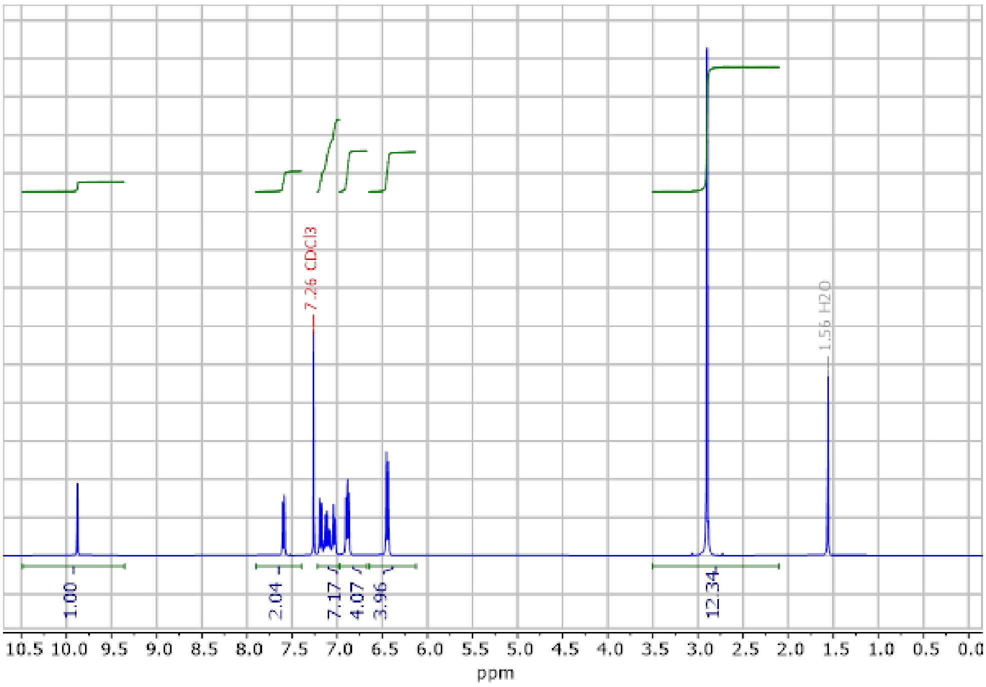

Figure S6: ^{1}H NMR spectrum (400 MHz, $CDCl_3$, 25°C) of TPE-CHO.

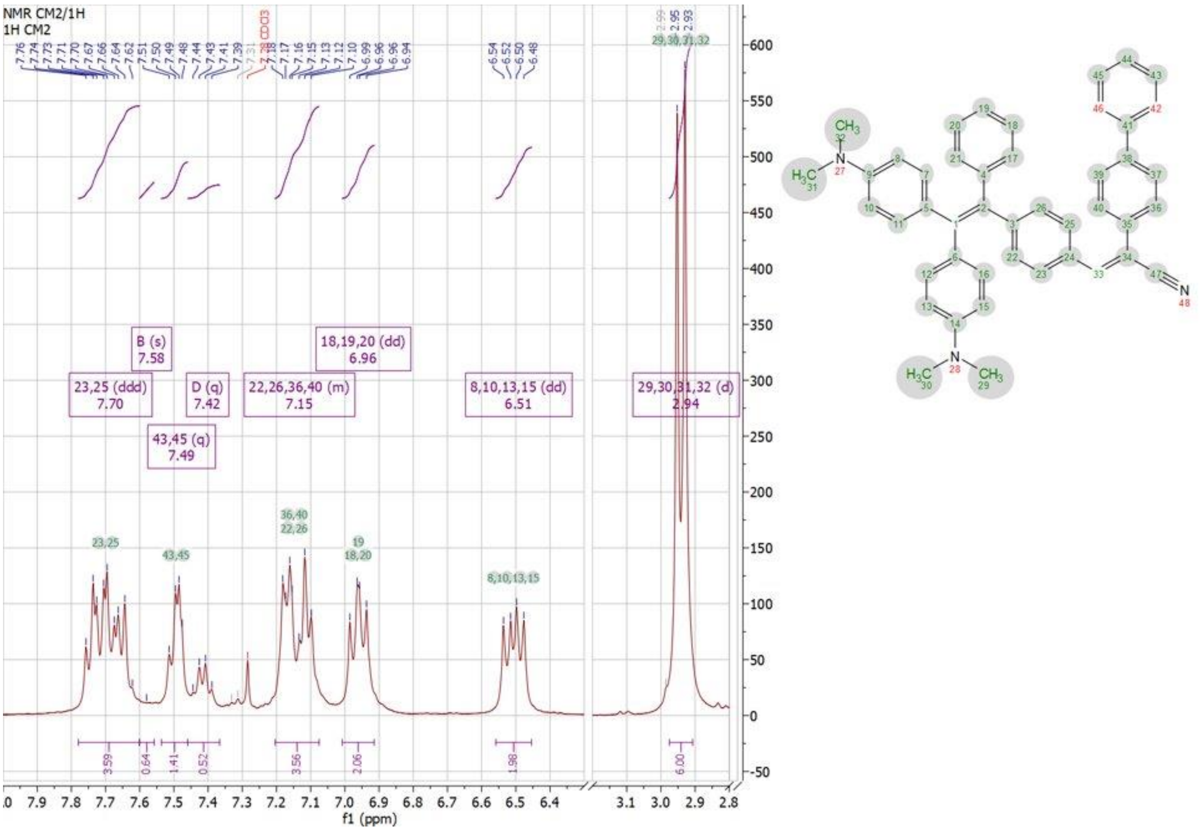

Figure S7: ¹H NMR spectrum (400 MHz, $CHCl_3$, 25°C) of TPE-BPAN.

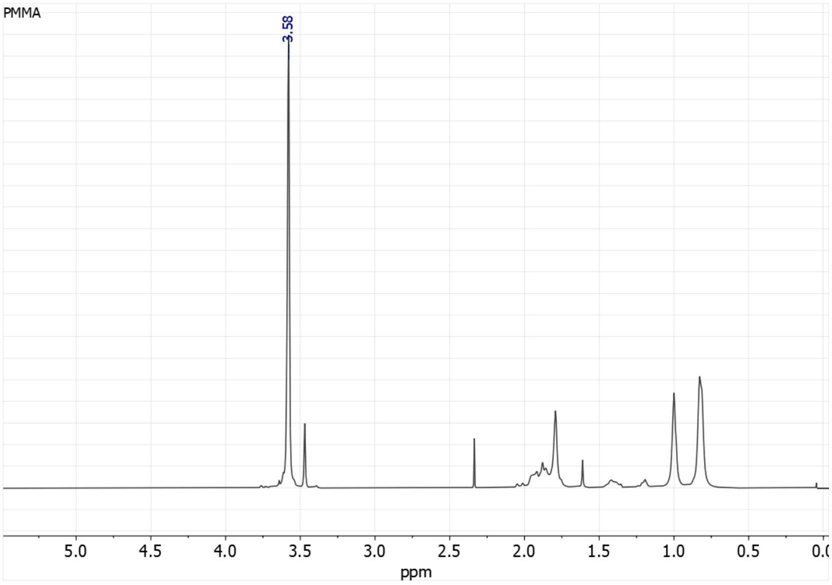

Figure S8: ¹H NMR spectrum (400 MHz, $CDCl_3$, 25°C) of PMMA.

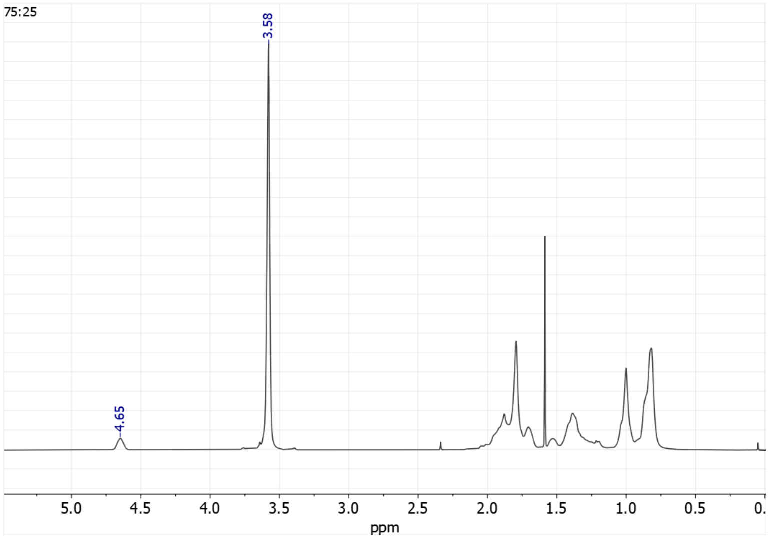

Figure S9: ^{1}H NMR spectrum (400 MHz, $CDCl_3$, 25°C) of P(MMA-co-CHMA) 75:25.

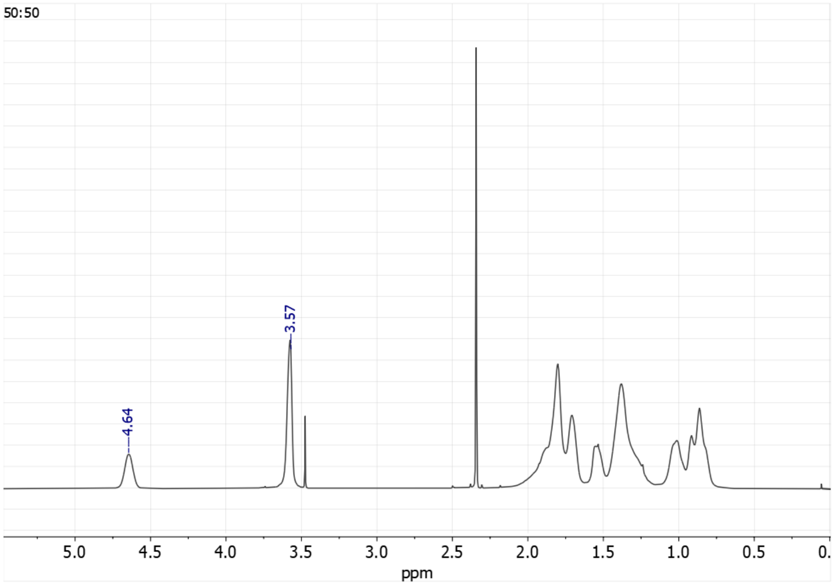

Figure S10: ^{1}H NMR spectrum (400 MHz, $CDCl_3$, 25°C) of P(MMA-co-CHMA) 50:50.

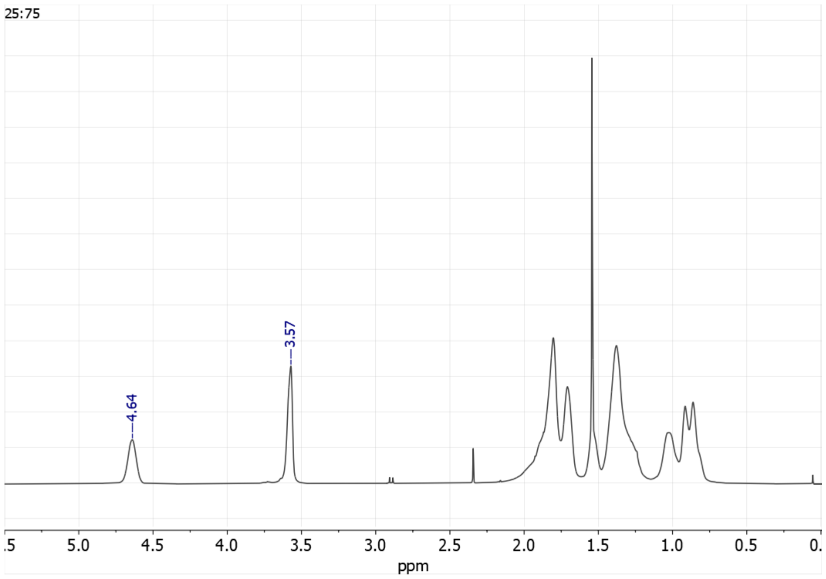

Figure S11: ^{1}H NMR spectrum (400 MHz, $CDCl_3$, 25°C) of P(MMA-co-CHMA) 25:75.

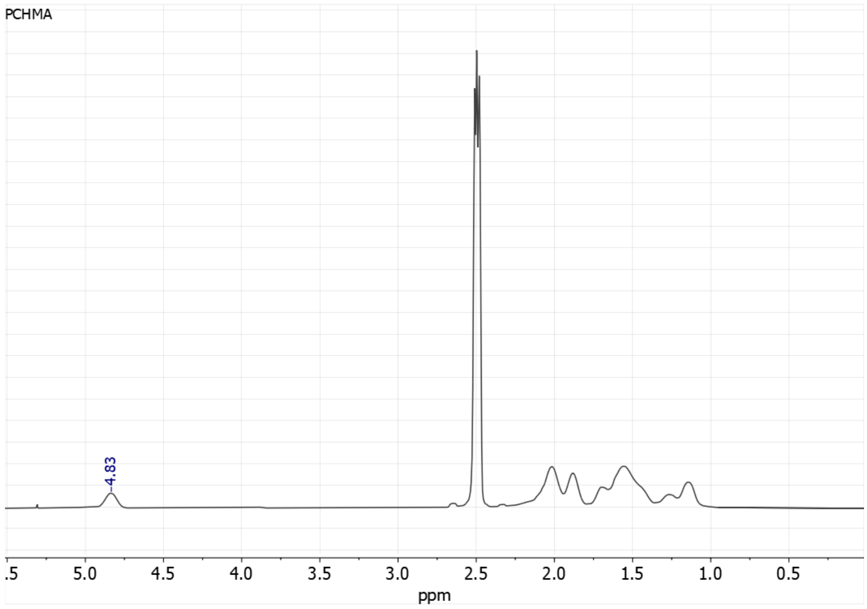

Figure S12: ^{1}H NMR spectrum (400 MHz, $CDCl_3$, 25°C) of PCHMA.

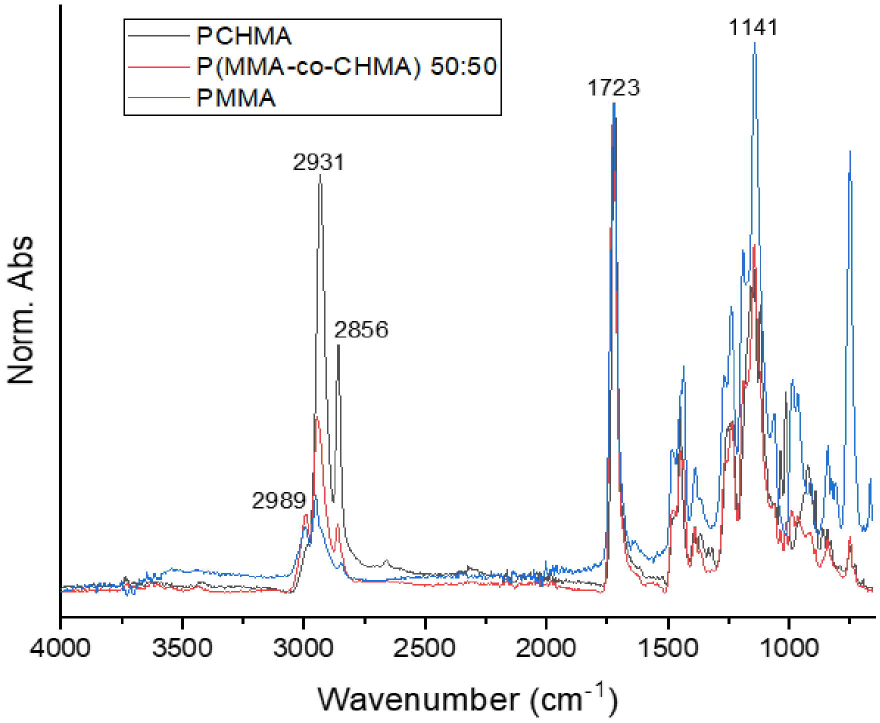

Figure S13: FTIR-ATR spectra of PMMA, P(MMA-co-CHMA) 50:50 and PCHMA samples. The position of peaks labelled correspond to: -CH- symmetrical and asymmetrical stretching (2989 cm^{-1}, 2931 cm^{-1}, 2856 cm^{-1}), -O-CH_3 stretching (1141 cm^{-1}). The reported spectra are normalized in relation to the intensity of C=O stretching (1723 cm^{-1}). No evident difference has been observed for co-polymers with different compositions.

GPC chromatograms

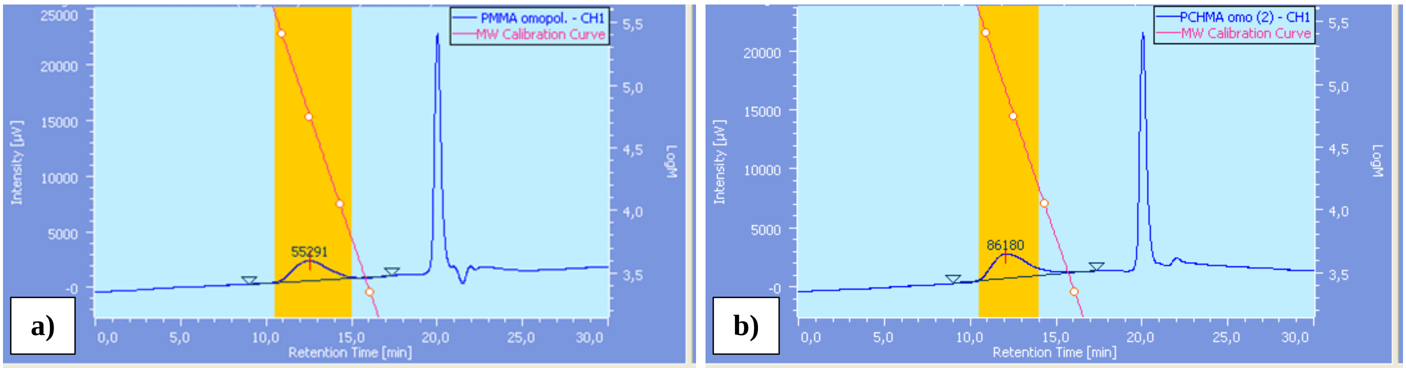

MW Calculation Results: PMMA omopol.

#	Range	CH	Baseline Range	tR	Mp	Mn	Mw	Mz	Mv	Mw/Mn
1	10,5000 - 15,0000	1	9,0000 - 17,3000	12,5333	55291	36343	67459	108296	67459	1,8562

MW Calculation Results: PCHMA omo (2)

#	Range	CH	Baseline Range	tR	Mp	Mn	Mw	Mz	Mv	Mw/Mn
1	10,5000 - 14,0000	1	9,0000 - 17,3000	12,0500	86180	52755	88157	132674	88157	1,6711

Figure S14: GPC chromatograms and MW calculation results of the two homopolymers synthesized. a) PMMA; b) PCHMA.

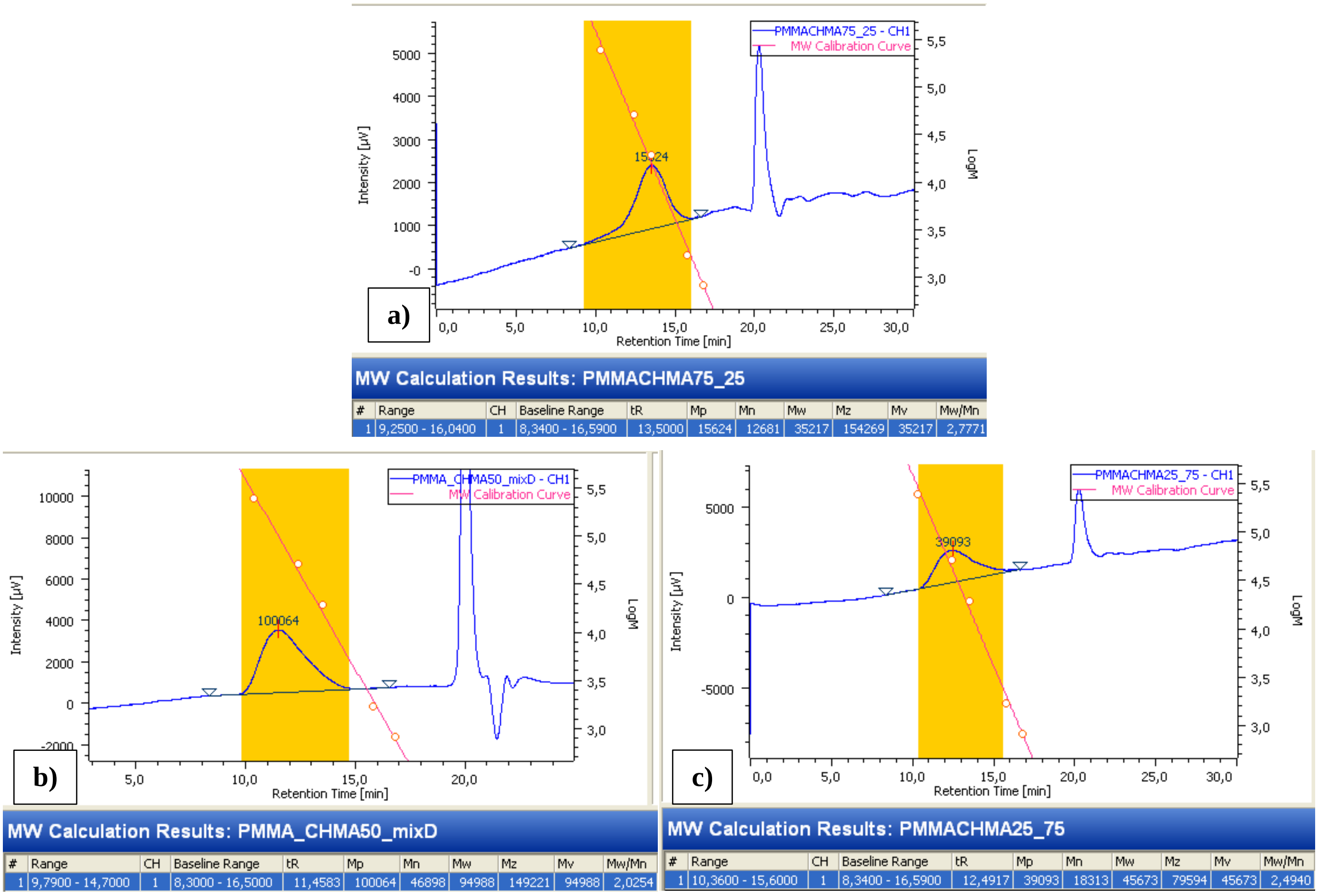

Figure S15: GPC chromatograms and MW calculation results of the three copolymers synthesized. a) P(MMA-co-CHMA) 75:25; a) P(MMA-co-CHMA) 50:50, c) a) P(MMA-co-CHMA) 25:75.

DSC

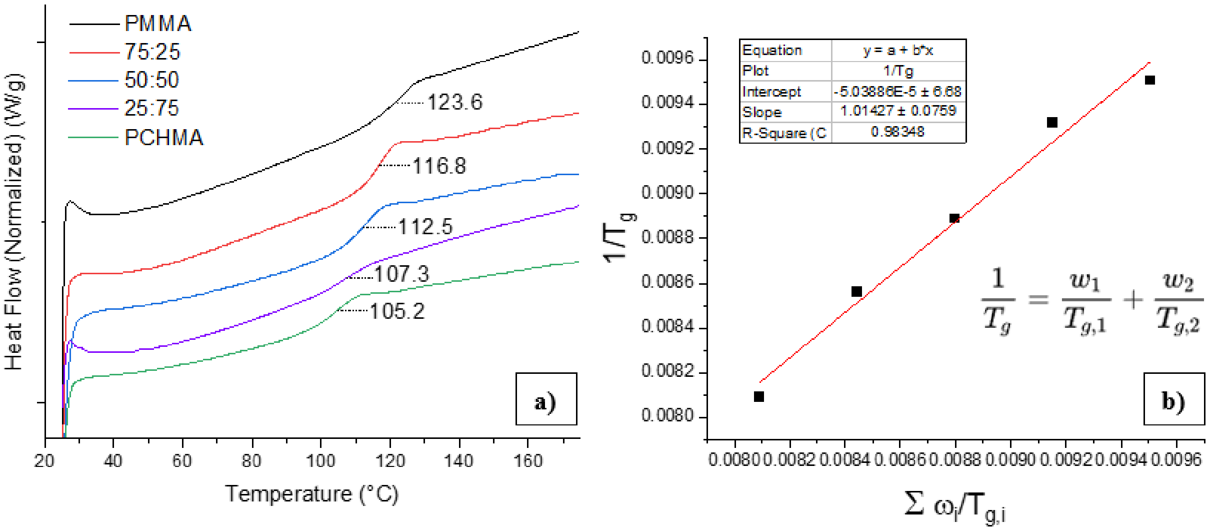

Figure S16: a) Superimposed DSC curves of the five polymeric matrices under study; b) Plot of the points relative to the five polymeric matrices and linear fitting of the Fox-Flory equation

Contact angle determination

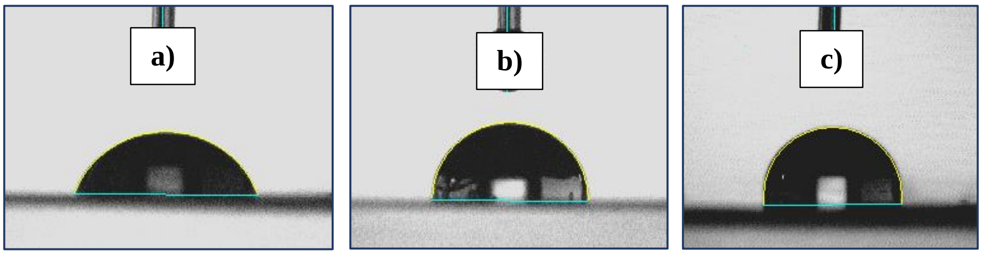

Figure S17: Contact angle pictures taken after 20 seconds after drop deposition on a) PMMA (68.8°); b) P(MMA-co-CHMA) 50:50 (90.2°); c) PCHMA (97.3°) films.

Epifluorescence images

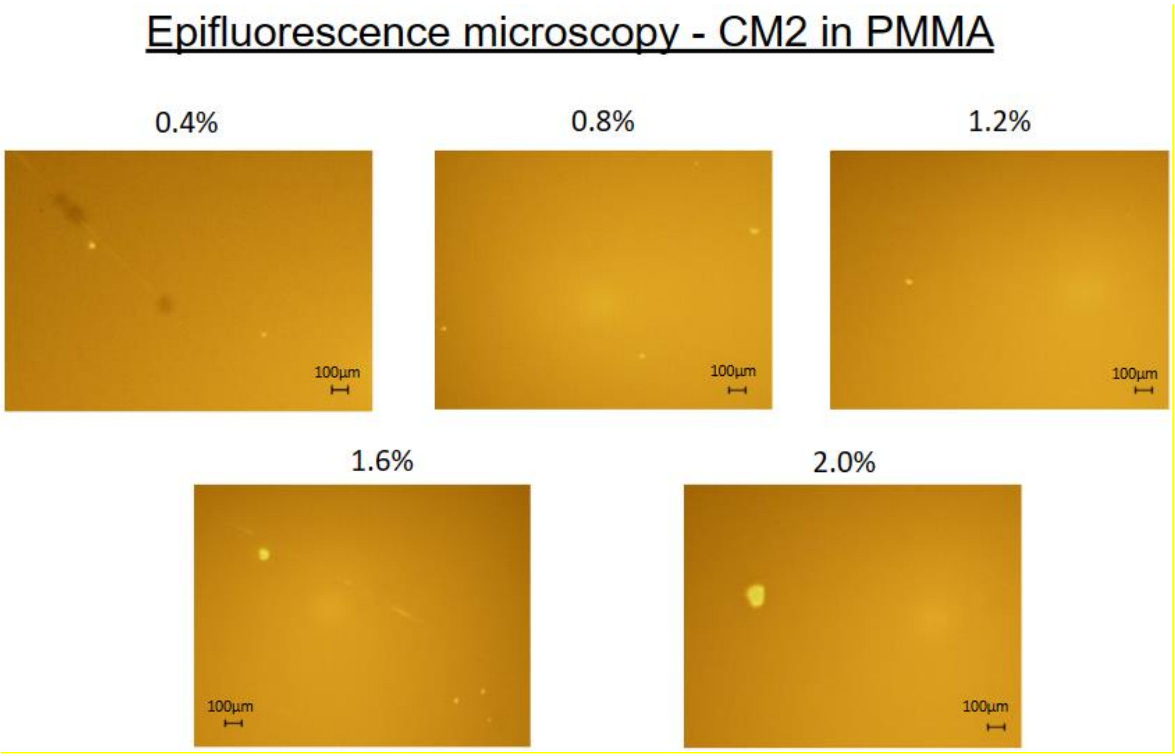

Figure S18: Epifluorescence microscopy images showing emitting fluorophore aggregates in PMMA) LSCs (scalebar = 100 µm)

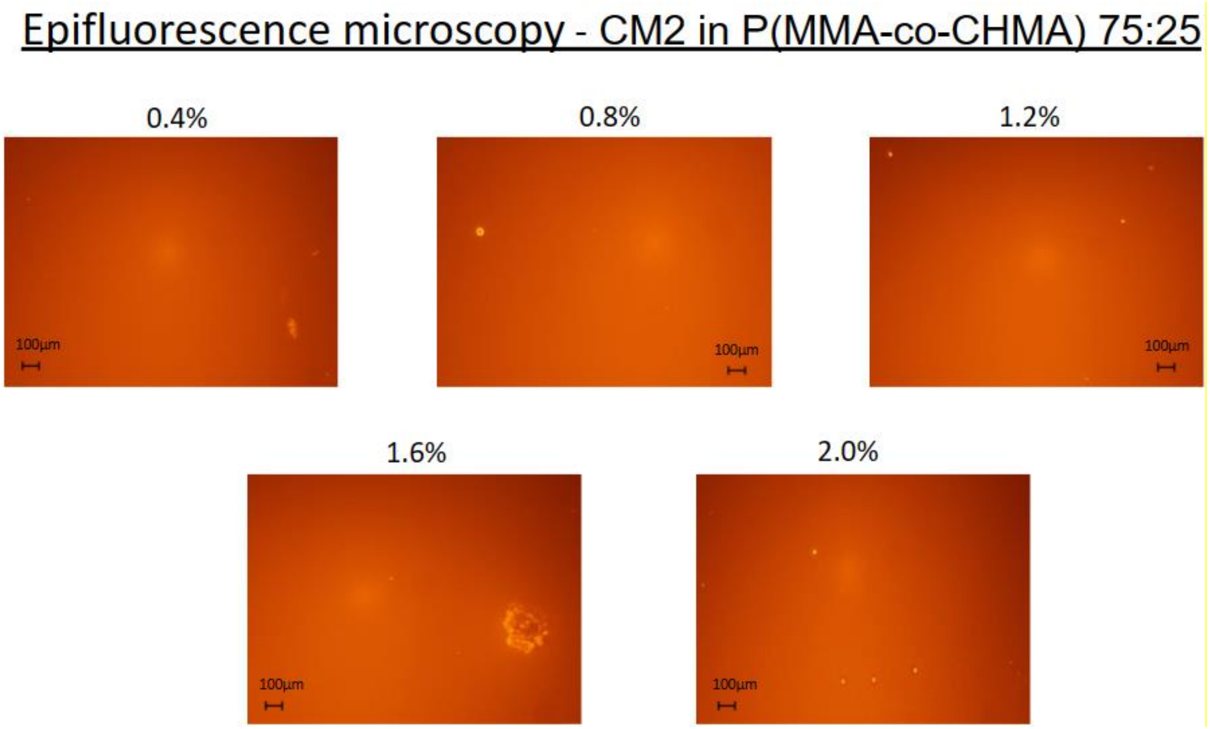

Figure S19: Epifluorescence microscopy images showing emitting fluorophore aggregates in P(MMA-co-CHMA) 75:25 LSCs (scalebar = 100 µm)

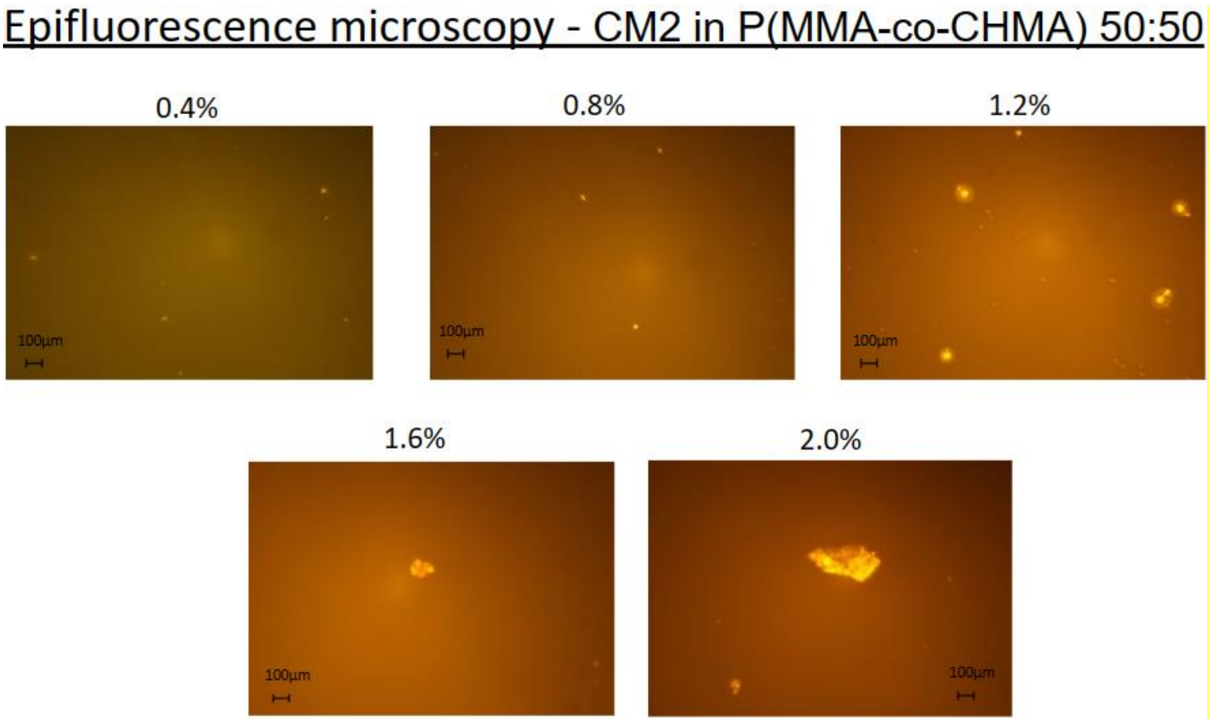

Figure S20: Epifluorescence microscopy images showing emitting fluorophore aggregates in P(MMA-co-CHMA) 50:50 LSCs (scalebar = 100 µm)

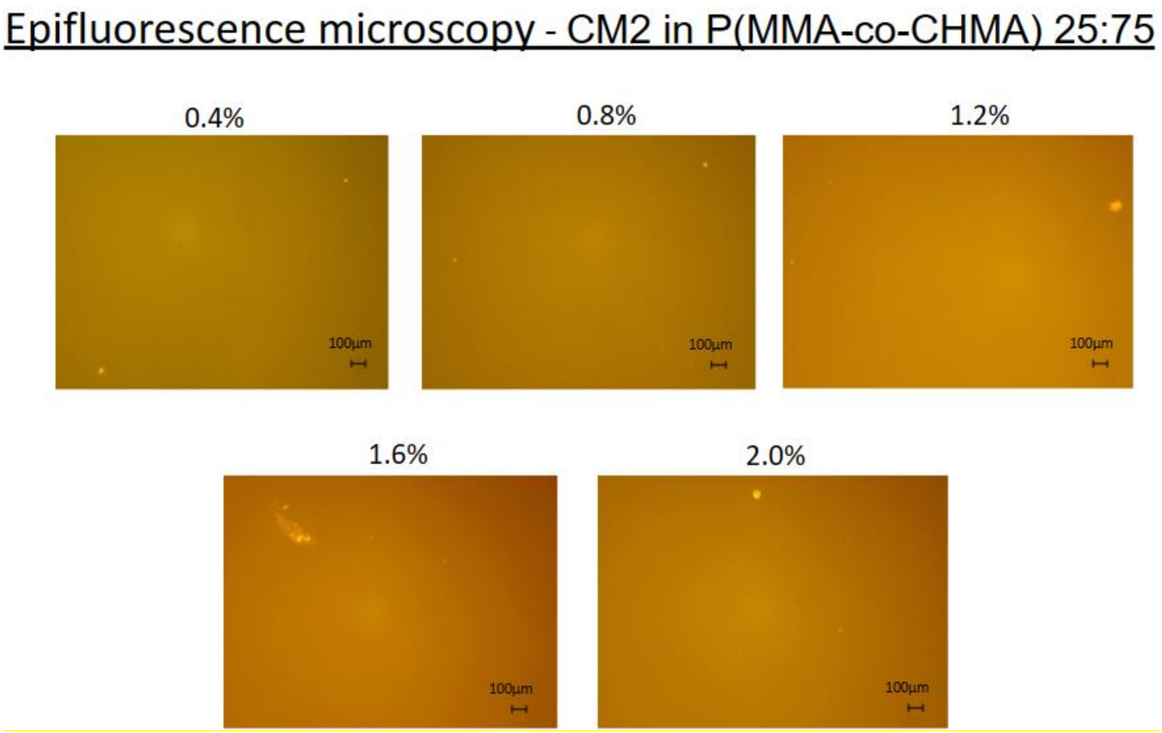

Figure S21: Epifluorescence microscopy images showing emitting fluorophore aggregates in P(MMA-co-CHMA) 25:75 LSCs (scalebar = 100 µm)

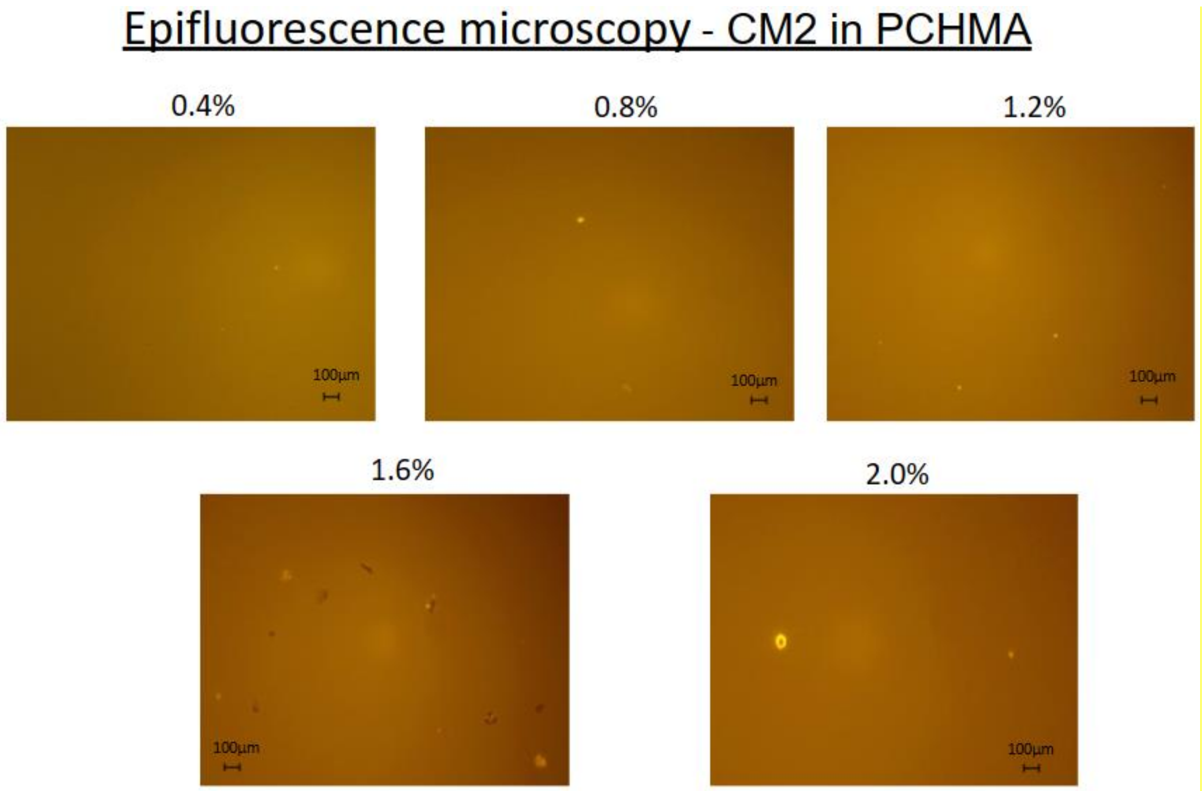

Figure S22: Epifluorescence microscopy images showing emitting fluorophore aggregates in PCHMA LSCs (scalebar = 100 µm)

Fluorophore synthesis

All reactions were performed with Schlenk techniques under a dry nitrogen atmosphere. THF, DCM, and Toluene were purified by a drying columns system (MBRAUN SPS-5). DMF anhydrous (Sigma-Aldrich) was used as received. Other solvents were directly used without further purification. The reagents, 4,4’-bis(dimethylamino)benzophenone (1), 4-bromobenzophenone (2), zinc, titanium(IV) chloride, n-butyl lithium (1.6 M in hexane), piperidine, and 4-biphenyl acetonitrile were purchased from Sigma-Aldrich and directly used without purification. Sigma-Aldrich precoated silica gel aluminum foils were used for TLC analyses. For analysis in solution, spectroscopic grade solvents were employed. Chromatographic separation was performed by using an automatic flash chromatography instrument (Biotage Isolera One) equipped with an UV-Vis detector and Biotage Sfär Silica D cartridge, loaded with 25 g or 50 g of 60 µm of silica gel.

Synthesis of 4,4'-(2-(4-bromophenyl)-2-phenylethene-1,1-diyl)bis(N,N-dimethylaniline) (TPE_Br)

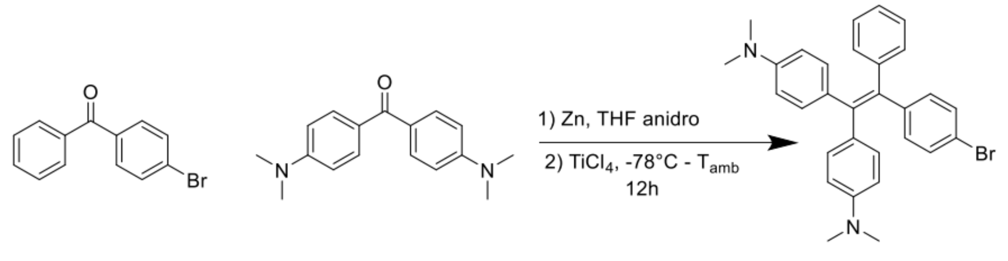

1 2 3

Figure S23: Reaction scheme of the synthesis of TPE_Br

According to a literature procedure[1,2], in a 250 mL dried two-necked flask 4,4’-bis(dimethylamino)benzophenone (1) (2.601 g, 9.7 mmol, 1 eq.), 4-bromophenyl)(phenyl)methanone (2) (3.312 g, 12.7 mmol, 1.3 eq.) and Zn (dust) (3.660 g, 56.0 mmol, 5.8 eq.) were suspended. 75 mL of anhydrous THF were added. The resulting mixture was cooled to −78 °C in a liquid nitrogen-ethyl acetate bath and $TiCl_4$ (3.6 mL, 6.23 g, 32.8 mmol, 3.4 eq.) was added dropwise. The mixture was then slowly warmed to room temperature, and subsequently stirred for 8 hours at reflux. After one night of stirring at room temperature, DCM (200 mL) was added and the zinc in excess filtered. Aqueous $NaHCO_3$ (10 wt.%) was added gently, the mixture was extracted, and the aqueous phase back-extracted with DCM (3 x 30 mL). The organic phase was dried over anhydrous $MgSO_4$, filtered, and concentrated at reduced pressure. The residue was purified with a silica gel chromatographic column using ISOLERA instrument using hexane/ethyl acetate/Et_3N (1%) for linear gradient eluting (85:15 v/v). The chromatographic fractions containing the required product were collected and concentrated at reduced pressure to afford product 4 as yellow solid (1.238 g, 2.47 mmol, isolated yield 25.5 %).

^{1}H-NMR (400 MHz, $CDCl_3$) δ (ppm): 7.24 – 7.15 (m, 2H), 7.15 – 6.97 (m, 6H), 6.95 – 6.75 (m, 5H), 6.59 – 6.32 (m, 4H), 2.91 (s, 6H), 2.88 (s, 6H).

Synthesis of 4-(2,2-bis(4-(dimethylamino)phenyl)-1-phenylvinyl)benzaldehyde (TPE_CHO)

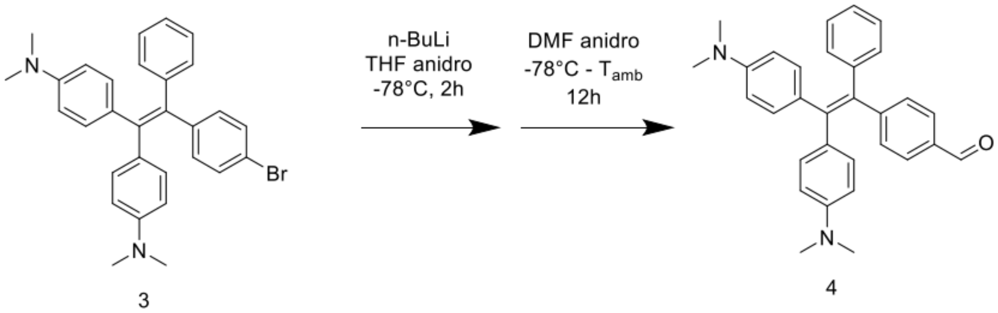

Figure S24: Reaction scheme of the synthesis of TPE_CHO

According to a literature procedure, 1,2 4,4'-(2-(4-bromophenyl)-2-phenylethene-1,1-diyl)bis(N,N-dimethylaniline) (3) (628 mg, 1.26 mmol, 1 eq.) anhydrous THF (22 mL) were added in a 100 mL dried two-necked flask. The mixture was cooled to –78 °C in a liquid nitrogen-ethyl acetate bath, and 1 mL of n-BuLi solution (1.6 M in hexane, 1.6 mmol, 1.27 eq.) was slowly added. The solution was kept under stirring at -78 °C for 2 h, after that 1 mL of anhydrous DMF (12.9 mmol, 10.2 eq.) was added, and the mixture was slowly recovered to room temperature and kept under stirring 2h. The reaction was quenched with 25 mL of saturated NaCl solution, the mixture was extracted, and the aqueous phase back-extracted with DCM (3 x 20 mL). The organic phase was dried over anhydrous $MgSO_4$, filtered, and concentrated at reduced pressure. The residue was purified with a silica gel chromatographic column using ISOLERA instrument with hexane/ethyl acetate (linear gradient, start 97:3, end 70:30) as the eluent. The chromatographic fractions containing the required product were collected and concentrated at reduced pressure to give 4 as an orange solid (159 mg, 0.36 mmol, isolated yield 28.6%). Its spectral properties agreed with those previously reported.

^{1}H-NMR (400 MHz, $CDCl_3$) δ 9.88 (s, 1H), 7.84 – 7.47 (m, 2H), 7.22 – 6.97 (m, 7H), 6.95 – 6.76 (m, 4H), 6.53 – 6.16 (m, 4H), 2.99 – 2.80 (m, 12H).

Synthesis of [(E)-2-([1,1'-biphenyl]-4-yl)-3-(4-(2,2-bis(4-(dimethylamino)phenyl)-1-phenylvinyl)phenyl) acrylonitrile] (TPE-BPAN)

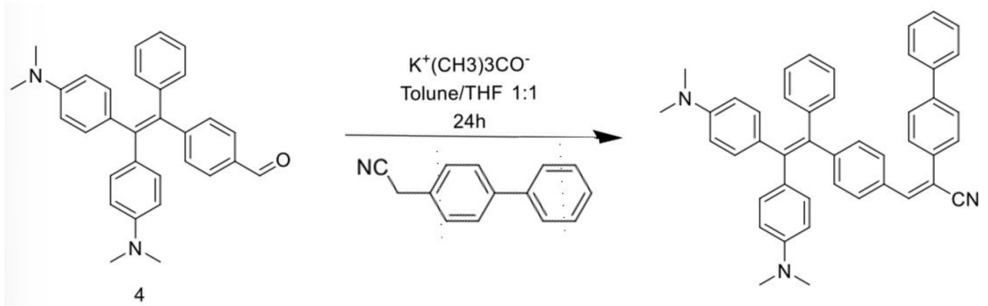

Figure S25: Reaction scheme of the synthesis of TPE-BPAN

In a 50 mL dried two-necked flask 95 mg of 4 (0.21 mmol, 1 eq.) and 66 mg of 4-biphenylacetonitrile (0.34 mmol, 1.6 eq.) were dissolved in 20 mL of 1:1 anhydrous toluene and absolute ethanol solution. Therefore, 68 mg of potassium tert-butyl oxide (61 mmol) were added to the reaction flask and the reaction was kept under stirring for 12 h at and for one night at room temperature. During the reaction a color change from yellow to orange was observed

(错误!未找到引用源。). The reaction mixture was washed with deionized water (3 x 20 mL), and the aqueous phase was back-extracted with DCM (3 x 20 mL). The organic extract was dried over anhydrous $MgSO_4$, filtered, and concentrated at reduced pressure. The residue was purified by silica gel chromatographic column using ISOLERA instrument, employing DCM/hexane for linear-gradient eluting (12:1 v/v). The chromatographic fractions containing the required product were collected and concentrated at reduced pressure to give TPE-BPAN as an orange solid (55 mg, 0.29 mmol, isolated yield 42.0%). The product was characterized by NMR spectroscopy, elemental analysis, and UPLC-MS.

^{1}H-NMR (401 MHz, $CDCl_3$) δ 7.87 – 7.58 (m, 8H), 7.57 – 7.35 (m, 4H), 7.23 – 7.06 (m, 7H), 7.05 – 6.83 (m, 4H), 6.51 (m, 4H), 3.21 – 2.58 (m, 12H).

^{13}C-NMR (100 MHz, $CDCl_3$) δ 149.17, 149.00, 148.57, 144.89, 143.07, 142.06, 141.73, 140.16, 136.03, 133.95, 132.84, 132.80, 132.27, 131.79, 130.78, 130.20, 129.05, 128.95, 127.95, 127.89, 127.74, 127.15, 126.35, 126.01, 118.52, 111.64, 111.46, 109.25, 40.56.

Elemental analysis calculated for $C_{45}H_{39}N_3$: C, 86.92; H, 6.32; N, 6.76; C/N, 12.86; C/H, 13.75. Found: C, 80.74; H, 5.99; N, 5.95; C/N, 12.57; C/H, 13.48.

ESI-MS (m/z) calculated for $C_{45}H_{39}N_3$: 621.31. Found: 311.8 $[M+2H]^{2+}$ and 621.5 $[M•]^{+}$.

Polymer syntheses

Methyl methacrylate (MMA, Aldrich) and cyclohexyl methacrylate (CHMA, Aldrich) were distilled before use. Inside a Carius tube, under a nitrogen atmosphere, the monomers were dissolved in toluene. After degassing the reaction medium with a stream of nitrogen, the initiator (AIBN, Aldrich) was added. The temperature was raised to 80°C for 24 to 72 hours to bring the conversion closer to unity. At the end, cold methanol was added dropwise while stirring. The white precipitate was filtered and dried at room temperature.

Table S1: Quantities of reagents, initiator and solvent used in the polymer syntheses.

Pol. No.	MMA addition	CHMA addition	Initiator	Solvent	Polymer weight
1	4.905 g, 0.048 mol	-	48.2 mg	≈ 15 mL	4.570 g
2	3.398 g, 0.034 mol	1.856 g, 0.011 mol	45.1 mg	≈ 15 mL	4.478 g
3	1.926 g, 0.019 mol	3.245 g, 0.019 mol	51.4 mg	≈ 15 mL	4.416 g
4	0.842 g, 0.007 mol	4.104 g, 0.024 mol	82.1 mg	≈ 15 mL	4.131 g
5	-	5.021 g, 0.030 mol	49.6 mg	≈ 15 mL	4.497 g

Thin-film deposition

Polymer thin films with a thickness of 25 ± 5 μm and containing TPE-BPAN were prepared by pouring 1.5 mL of $CHCl_3$ solution containing about 60 mg of the polymer and different concentrations (0.4–2.0 wt.%) of the fluorophore on a 50 x 50 x 3 mm^3 optically pure glass substrate (Edmund Optics Ltd BOROFLOAT window 50x50 TS)[3].

Thin-film characterization

NMR spectra were recorded at room temperature at 400 MHz (^{1}H-NMR) and 100 MHz (^{13}C-NMR) on a Bruker Avance DRX 400 spectrometer using $CDCl_3$ as solvent, and the residual solvent peak as internal standard.

FT-IR spectra were acquired by a Nicolet iS50 spectrometer (ThermoFisher). The samples were analyzed in the ATR mode in the spectral range between 4000 and 600 cm^{-1}. The ATR accessory (ATR ITX) contained a diamond crystal (by employing this crystal, the penetration of infrared radiation is about 2 μm at wavelength numbers 1000 cm^{-1}) at a nominal incident angle of 45°.

UPLC-MS analysis was performed on an Acquity UPLC Water instrument (Phase A 95/5 H_2O/MeCN + 0.1% Formic Acid, Phase B 5/95 H_2O/MeCN + 0.1% Formic Acid; Acquity UPLC 2.1x100 mm column, BEH C18, 1.7 μm; Flow 0.6 mL/min) coupled with an Acquity QDa Water mass spectrometer (Probe temperature: 600 °C; ESI capillary voltage 1.5V; Cone voltage 15V; Mass range 60-1000).

Elemental composition was determined by using an Elementar Vario Micro Cube for nitrogen, carbon, sulfur, and hydrogen. UV-Vis absorption spectroscopy was performed using a Cary 5000 UV-Vis-NIR spectrophotometer (Agilent) equipped with liquid or solid sample holder. Fluorescence spectra were measured using a Fluorolog-3 spectrofluorometer (Horiba) with an integration time for the analysis between 0.1 and 0.3 s. The selected excitation wavelengths were close to the absorption peak. For polymeric films, the samples were rotated 30 degrees relative to the excitation beam, and the detector was set in front-face mode. Quantum yield measurements were carried out using an external integration sphere (Quanta-ϕ F-3029, Horiba) equipped with a solid sample holder, and connected to the spectrofluorometer by optical fibers and a fiber-optics adaptor (FL-3000, Horiba).

Thermogravimetric Analysis (TGA) was carried out on with a Mettler Toledo TGA/SDTA 851 instrument at a heating rate of 10 °C/min from room temperature to 700 °C, with as the purge gas (60 mL/min). X-ray powder diffraction using a Bruker D2 Phaser diffractometer (30 kV, 10 mA) operating in Bragg-Brentano geometry (θ-θ scan mode) and equipped with a 1-dimensional Lynxeye detector. Ni-filtered Cu Kα radiation was used. Data were collected in the scan range 5-40° in 2θ, with scan step of 0.02° and counting times of 0.1 s/step. Data were processed through the software Diffrac. Eva (Bruker AXS). Static contact angle measurements were carried out on PMMA and PCHMA polymer films with a FTA200 Camtel goniometer using water (J. T. Baker, Center Valley PA, HPLC grade). TPE-BPAN aggregates was measured in a THF/water mixture (20/80 by vol.%) by means of a Beckman-Coulter DelsaTM Nano C dynamic light scattering at 25°C.

All measurements to determine photonic and device efficiencies were performed by using a commercially available system (Arkeo – Cicci research s.r.l.) containing a CMOS-based spectrometer with a symmetrical Czerny-Turner optical bench connected to an integrating

sphere. As illumination source an ORIEL® LCS-100 solar simulator 94011A S/N: 322 was utilized under controlled illumination (1 sun, AM 1.5G). An integrating sphere of 5 cm of diameter and 1 cm of aperture is placed along the edge of the glass plate. To avoid the collection of the stray light, the sphere was covered by an opaque plastic holder with a rectangular aperture of 1 cm (the diameter of the sphere) x 3 mm (i.e., the thickness of the LSC slab). The spectrally-resolved edge output photon count was collected from the CMOS-based spectrometer and calibrated into optical power (W) and then in irradiance. Aimed at limiting reflections of unabsorbed light, an absorbing matte black background was placed in contact with the LSC rear side.

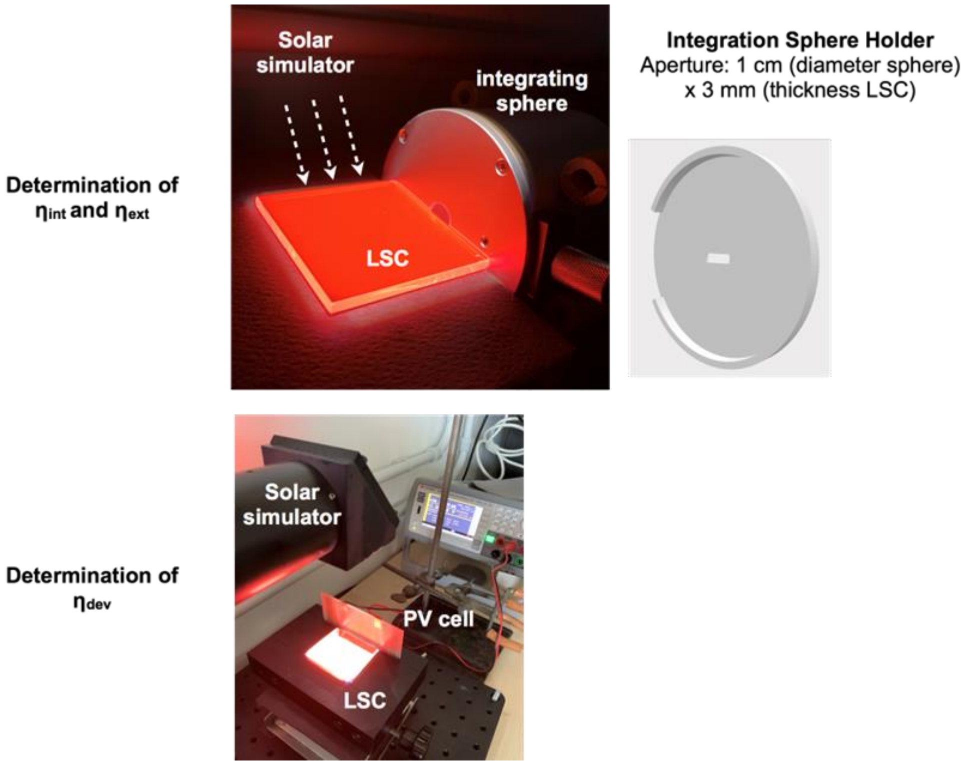

Figure S26: Photos of the experimental setup utilized for the determination of the photonic and device efficiencies

The optical performances of LSC were evaluated in terms of the internal and the external photon efficiency (η_{int} and η_{ext}, respectively). η_{int} and η_{ext} were calculated from the equations S1 and S2:

$$\eta_{int} = \frac{\text{no. of edge} - \text{emitted photons}}{\text{no. of total absorbed photons}} = \frac{\sum_{i=1}^{i=n} \int_{\lambda_1}^{\lambda_2} P_{out,i}(\lambda) \frac{\lambda}{hc} d\lambda}{\int_{\lambda_1}^{\lambda_2} P_{abs}(\lambda) \frac{\lambda}{hc} d\lambda} = \frac{\sum_{i=1}^{i=n} \int_{\lambda_1}^{\lambda_2} P_{out,i}(\lambda) \frac{\lambda}{hc} d\lambda}{\int_{\lambda_1}^{\lambda_2} (P_{in}(\lambda) - P_{tr}(\lambda)) \frac{\lambda}{hc} d\lambda} \quad \text{(Eq. S1)}$$

$$\eta_{ext} = \frac{\text{no. of edge} - \text{emitted photons}}{\text{no. of total incident photons}} = \frac{\sum_{i=1}^{i=n} \int_{\lambda_1}^{\lambda_2} P_{out,i}(\lambda) \frac{\lambda}{hc} d\lambda}{\int_{\lambda_1}^{\lambda_2} P_{in}(\lambda) \frac{\lambda}{hc} d\lambda} \quad \text{(Eq. S2)}$$

Where:

a) $n = 4$, $\lambda_1 = 300$ nm and $\lambda_2 = 1100$ nm;

b) the number of edge-emitted photons were obtained from the sum of the output power spectra measured for each edge of the LSC;

c) the number of total absorbed photons was obtained as shown in Eq.S1 from the absorbed power spectrum, derived from the difference between the power spectra of the incident light and the power transmitted by the LSC.

d) The total number of photons incident on the front surface of the LSC was obtained from the input power spectrum of the light source incident on the illuminated surface area of the LSC.

The concentration factor (C) can be eventually determined from the external photon efficiency and the LSC dimensions in agreement with the relation: $C = G\ \eta_{ext}$, where G is the geometrical gain defined as the ratio between LSC surface area and LSC edge area. In the case of a 5 x 5 x 0.3 cm^3 LSC, the geometric factor G measures 16.6.

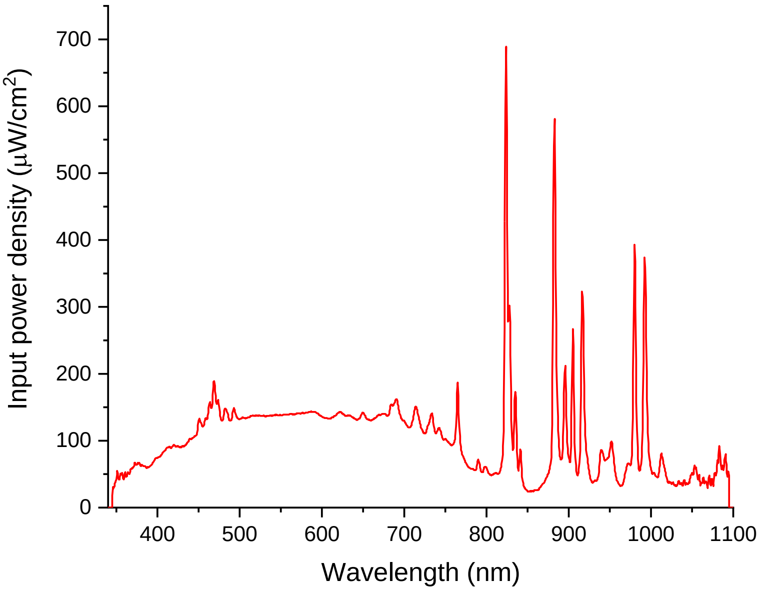

Figure S27: Input power density of the light source incident on the illuminated surface area of the LSC, Pin(λ)

The LSC efficiency is determined connecting two Si-PV cells in series to an edge of the thin-film LSCs by using silicone grease. The performance of the assembled LSC-PV systems is assessed under standard illumination conditions by measuring the power conversion efficiency of the resulting LSC device (η_{dev}), defined as the electrical power effectively extracted from the PV cells (P^{out}_{el}) relative to the luminous power hitting the top surface of the LSC (P^{in}_{opt}):

$$\eta_{dev} = \frac{P_{el}^{out}}{P_{opt}^{in}} = \frac{FF \cdot I_{SC} \cdot V_{OC}}{P_{opt}^{in} \cdot A_{LSC}} \text{ (Eq. S3)}$$

where FF, I_{SC}, and V_{OC} are the fill factor, short-circuit current, and open-circuit voltage of the edge-mounted PV cells, respectively, A_{LSC} is the front-illuminated area of the LSC device, and P^{in}_{opt} is the incident solar power density expressed in mW cm^{-2}.

For the determination of η_{dev}, two PV cells IXYS KXOB25-12X1F (22 x 7 mm, V_{oc} = 0.69 V, I_{sc} = 46.7 mA, FF > 70%, η = 25%) were connected in series and the current/voltage characteristics determined with a precision source/measure unit (Keysight Technologies B2900 Series). Silicon was used to grease the LSC edge. A black matte layer was placed beneath the LSC with an air gap of about 1.5 cm during the measurements.

Quantum yield

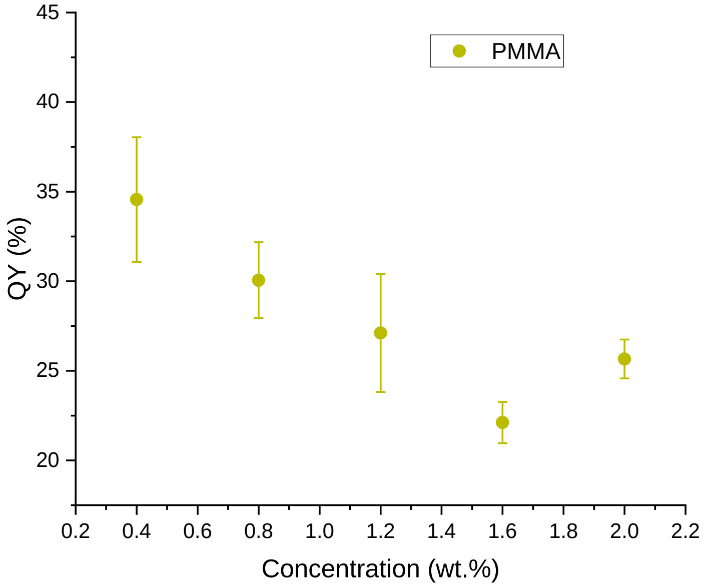

Figure S28: Fluorophore quantum yield (QY) vs. TPE-BPAN concentration in PMMA thin films.

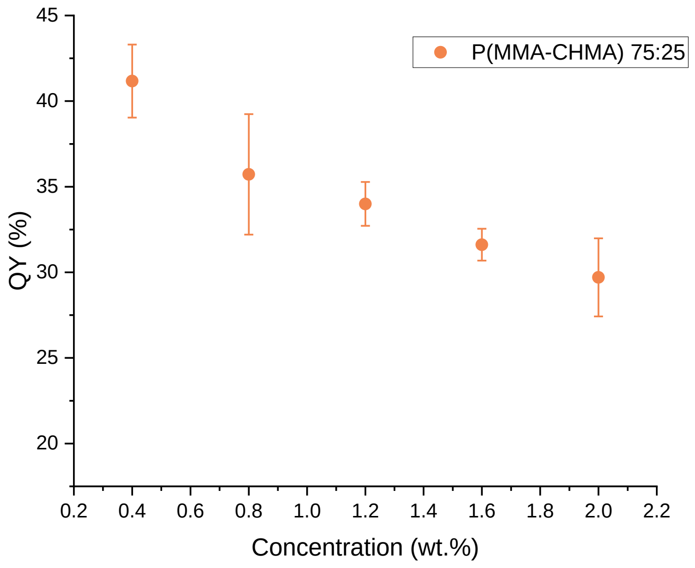

Figure S29: Fluorophore quantum yield (QY) vs. TPE-BPAN concentration in P(MMA-co-CHMA) 75:25 thin films.

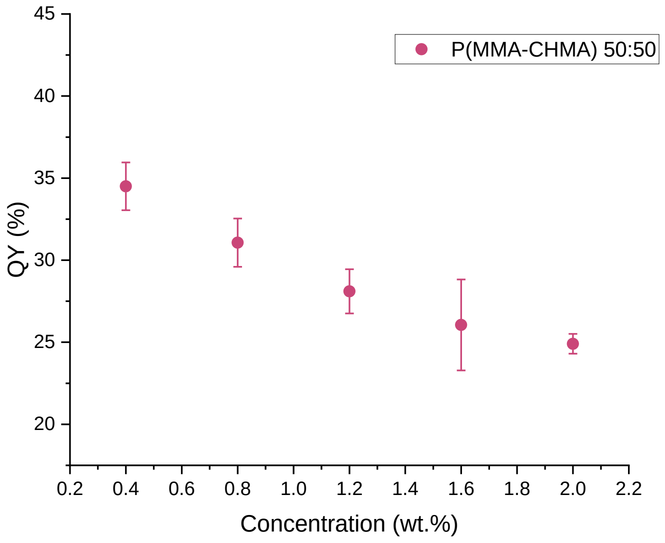

Figure S30: Fluorophore quantum yield (QY) vs. TPE-BPAN concentration in P(MMA-co-CHMA) 50:50 thin films.

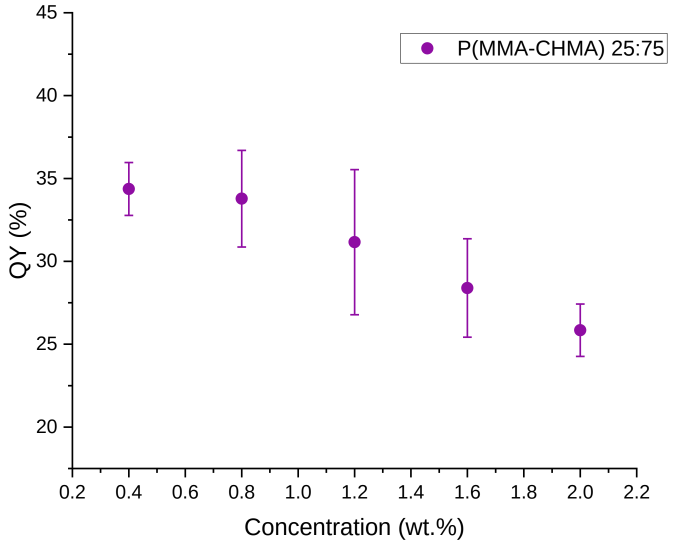

Figure S31: Fluorophore quantum yield (QY) vs. TPE-BPAN concentration in P(MMA-co-CHMA) 25:75 thin films.

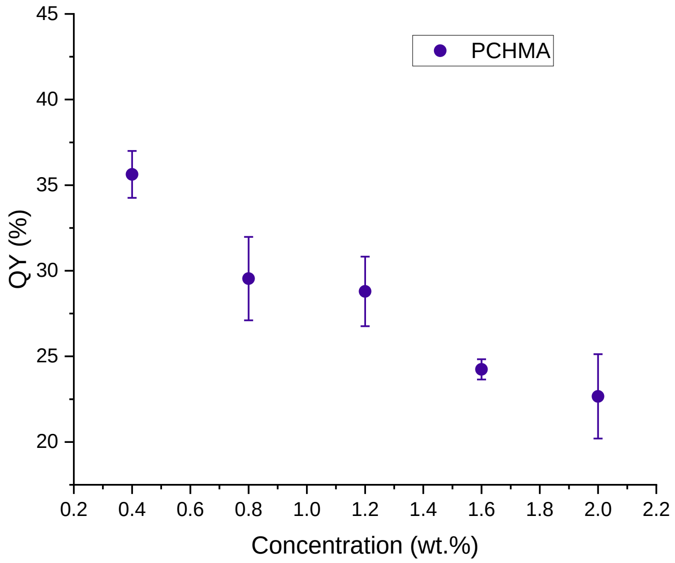

Figure S32: Fluorophore quantum yield (QY) vs. TPE-BPAN concentration in PCHMA thin films.

2. Photos

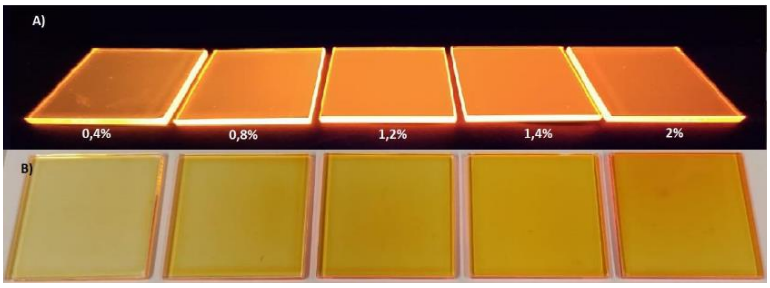

Figure S33: TPE-BPAN/PMMA thin-films at fluorophore concentrations from 0.4 wt.% to 2.0 wt.%.

3. Computational details

DFT and TD-DFT calculations were performed to describe ground and excited states, respectively. All calculations for molecular -non-periodic- systems were performed using the Gaussian 16 package[4]. Solvent (water or THF) was included implicitly via the polarizable continuum model (PCM)[5]

All data reported in the main text, unless differently stated, are performed using the global hybrid PBE0 exchange correlation functional.[6,7] For structural optimization, default force and the energy thresholds were applied and the nature of the so obtained structure as energy minima was confirmed by harmonic vibrational frequencies calculations performed on the final structures.

Structural optimization corresponding to data discussed in the main text were performed for ground (S_0) and excited states (S_n) using the 6-31G(d) and the 6-31+G(d), respectively.[8] An assessment of this level of theory for reproducing absorption and emission properties of TPE-BPAN in solution is provided below (SI section TPE-BPAN Absorption and Emission).

To assess the strength of the interaction between molecular vibrations and electronic degrees of freedom, which can be correlated to the efficiency of non-radiative de-excitation channels mediated by molecular motions, Huang-Rhys (HR) factors[9] were computed. These dimensionless factors are defined for each of the fundamental molecular normal modes according to the formula:

$$\mathrm{HR}_k = \frac{\Omega_k d_k^2}{2\hbar}$$

where W_k is the frequency of the *k*-th normal mode and d_k is the displacement of the minimum position between the ground and the target excited state. The computation of HR factors was carried out using Gaussian16 at the same level of theory described above.

TPE-BPAN Absorption and Emission

The performance of the global hybrid PBE0 in the prediction of absorption and emission spectra of TPE-BPAN in pure THF solution using the Linear Response (LR) TD-DFT approach was compared to the results obtained using a range separated hybrid (CAM-B3LYP)[10] considering the LR, the corrected linear response (cLR) and state-specific (SS) approaches.[11,12,13] In all cases, the 6-31+G(d) basis set was used. The absorption and emission energy corresponding to the lowest energy transition are collected in Table S2 in comparison with the available experimental data.

Generally, the range separated hybrid CAM-B3LYP is overestimated the absorption energy independently in the TD-DFT implementation while the global hybrid PBE0 in the LR TD-DFT implementation is underestimated it.

If the CAM-B3LYP(SS) approach is providing the best agreement with the experimental data for absorption energy it should be noted that the predicted Stokes shift at this level of theory is strongly overestimated due to a significant underestimation of the emission energy.

Consequently, for all further calculations we preferred to use the PBE0 LR TDDFT approach since it is providing a consistent description of the absorption and emission energies and thus a sufficiently accurate prediction of the Stokes shift of TPE-BPAN at a sustainable computational cost.

Table S2. Absorption and emission energies of TPE-BPAN computed in THF

System	Functional	Computed	Exp.
E_{abs} (eV)	CAM-B3LYP (LR)	3.351	2.793
	CAM-B3LYP (cLR)	3.263	
	CAM-B3LYP (SS)	2.815	
	PBE0 (LR)	2.281	
E_{emi} (eV)	CAM-B3LYP (LR)	1.713	2.006
	CAM-B3LYP (cLR)	1.753	
	CAM-B3LYP (SS)	1.524	
	PBE0 (LR)	1.578	

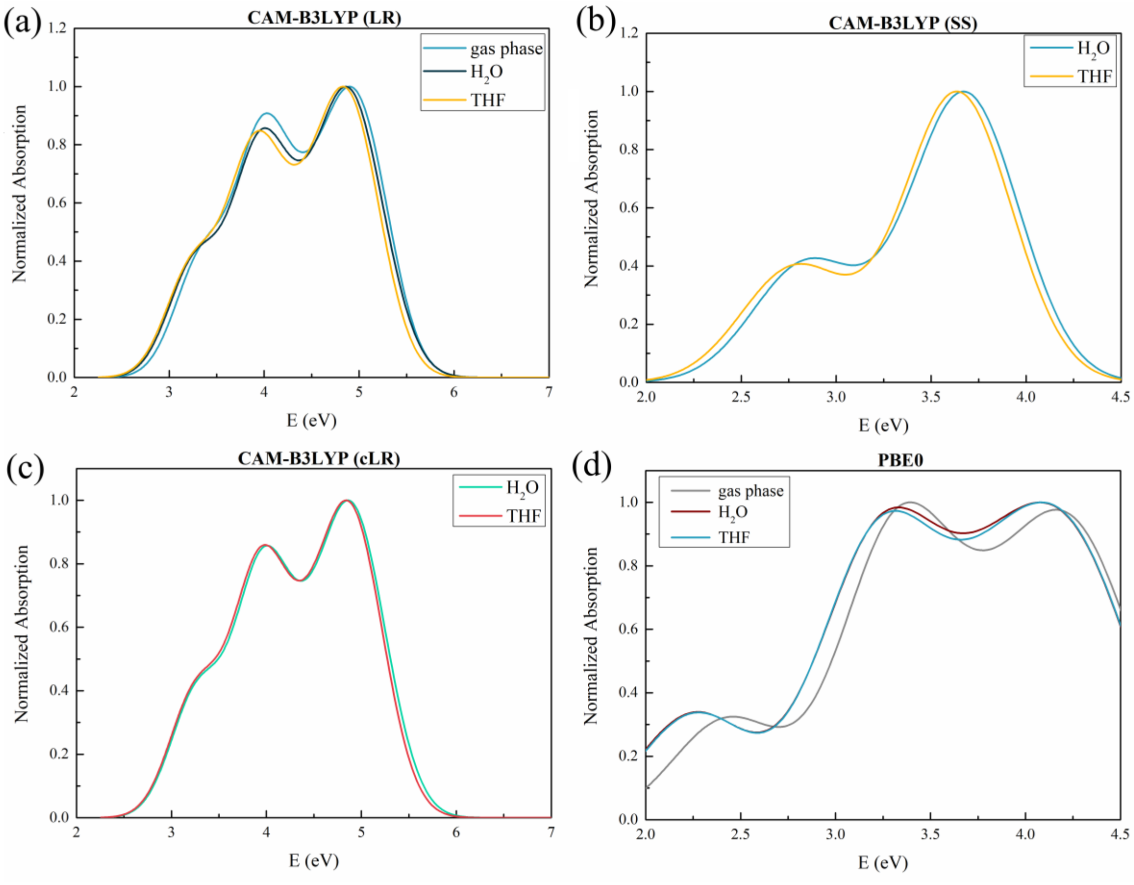

Figure S34:Computed absorption spectra.

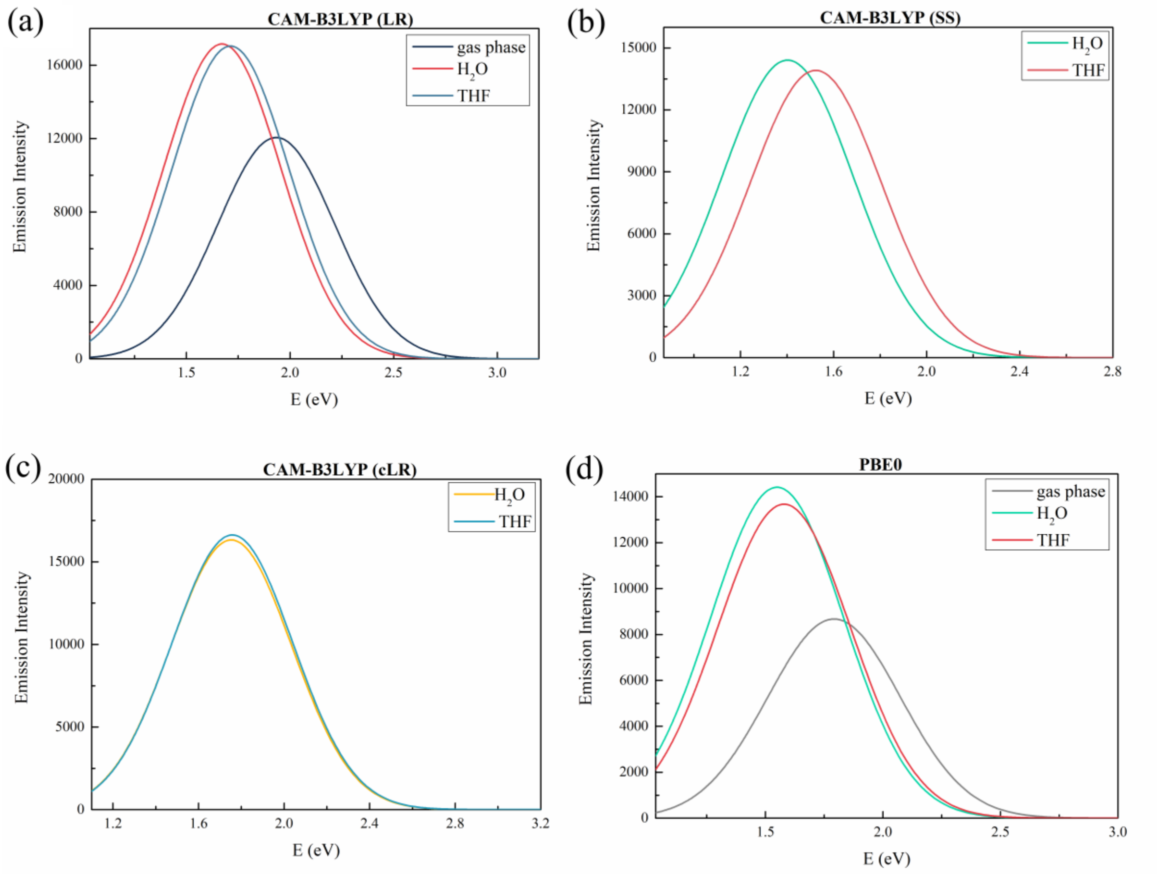

Figure S35: Computed Emission spectra.

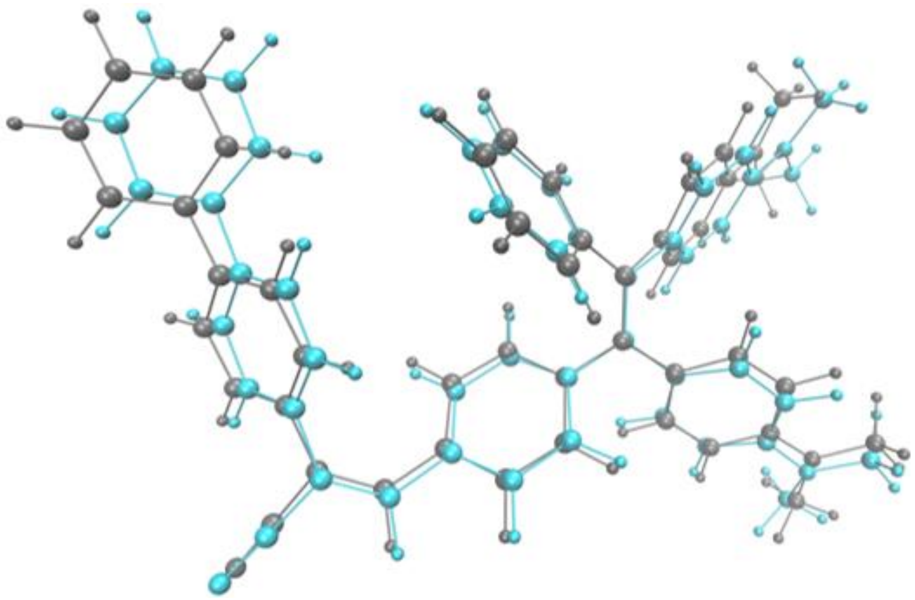

Figure S36: Superposition of the optimized ground state (S0, gray) and first excited state (S1, cyan) structures computed for TPE-BPAN

Table S3. Difference in dihedral angles of TPE-BPAN computed between the ground (S0) and first (S1) excited state structures (PBE0 level of theory in THF and water solution). The labelling scheme used is reported in the Figure below the table.

	H_2O	THF
Δα (°)	7.663	7.393
Δβ (°)	11.329	10.375
Δγ (°)	12.960	12.999
Δζ (°)	9.802	7.626
Δφ (°)	-14.129	-14.093
Δλ (°)	-16.805	-15.871
Δσ (°)	2.721	2.770
Δω (°)	-15.643	-14.948

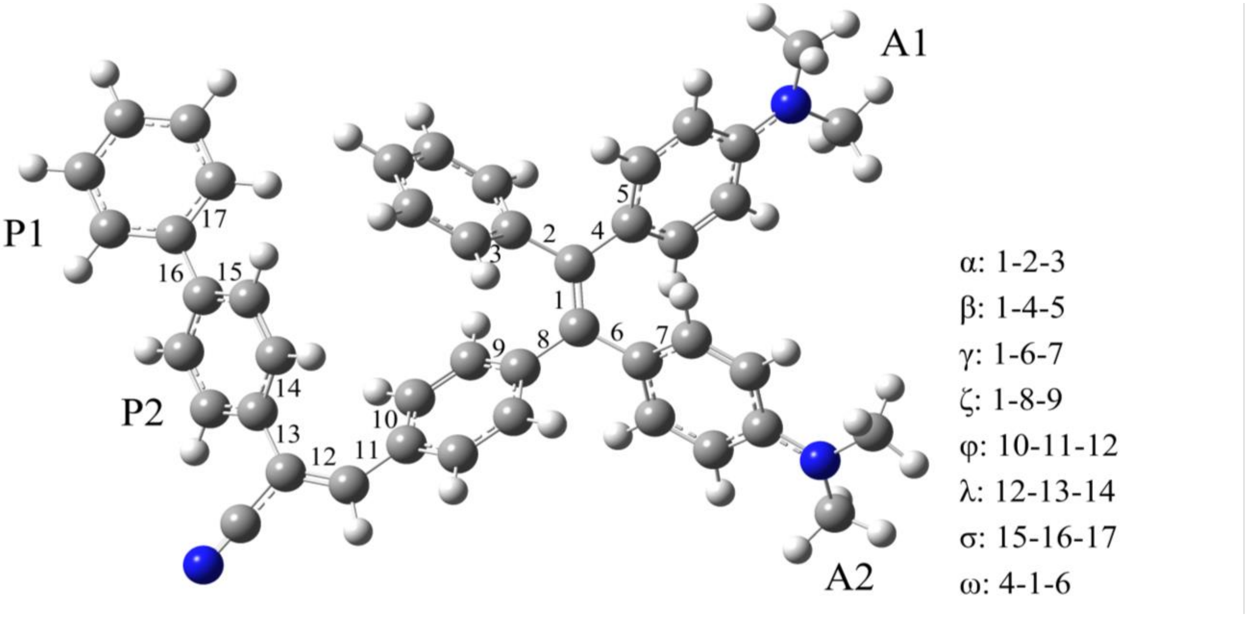

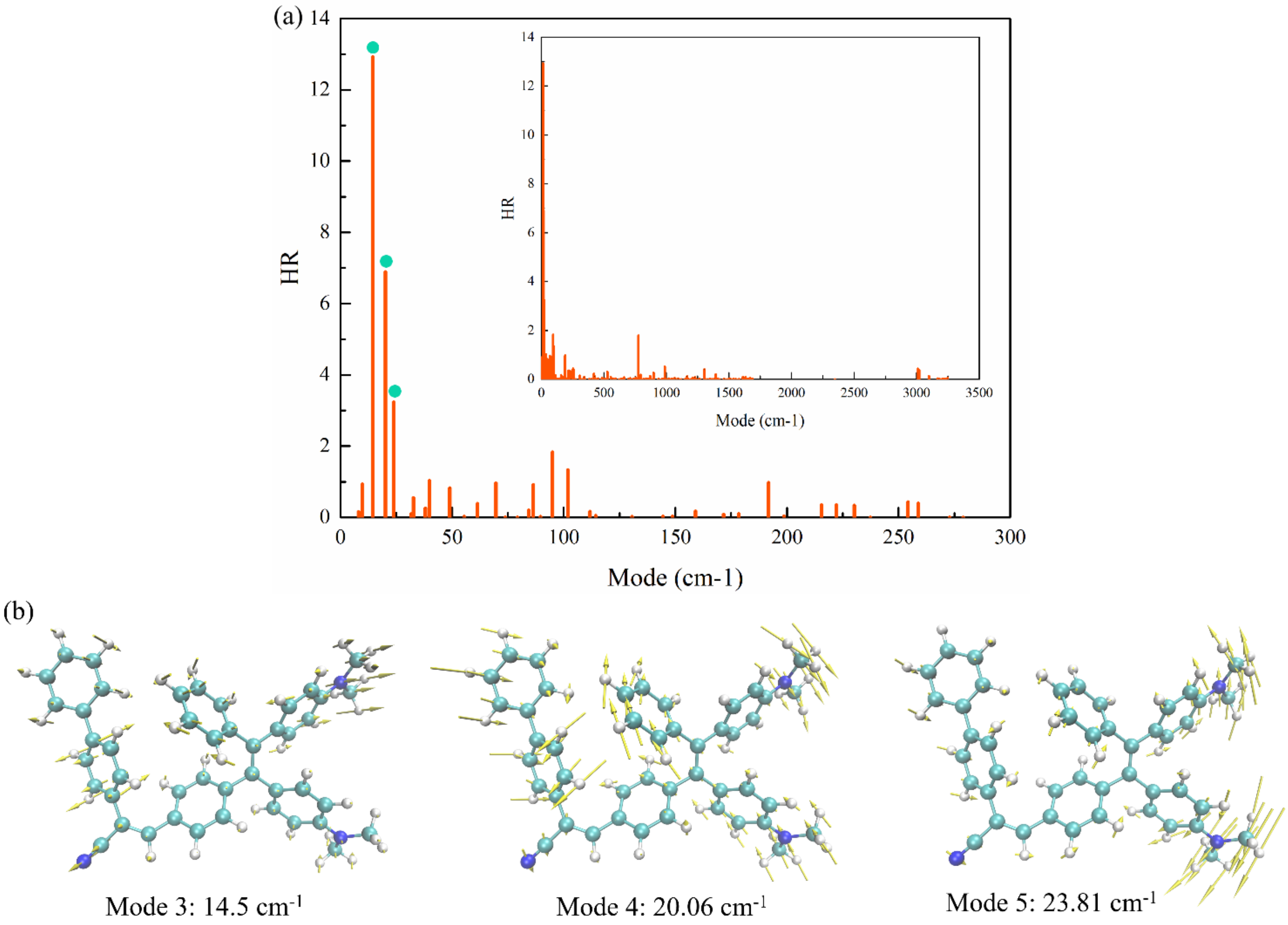

Figure S37. a) Calculated Huang-Rhys factors (HR) versus normal-mode wave number (ω, cm^{-1}) of TPE-BPAN for the S_0-S_1 transition in THF.HR b) schematic representation of normal modes corresponding to the three most intense HR factors.

TPE-BPAN crystals

Experimentally, the XRD pattern (see Figure S4) suggests that TPE-BPAN aggregates formed in solution upon addition of water to THF solution have predominant polycrystalline character analogously to the TPE-MRh case. To identify the most contributing polymorphs, a Monte Carlo sampling of possible polymorphs was performed using the dmol[3] and polymorph modules of the Materials Studio[14] software. To this end, we used the DREIDING force field[15] complemented with ESP charges[16] obtained from a B3LYP[17] calculation on the isolated molecule. For the polymorph search we restricted to the following space groups: $P2_{1/C}$, $P2_12_12_1$, $P2_1$, Pbca, Pbcn, $Pna2_1$. The three most stable polymorphs identified were then refined to better evaluate their structure and relative stability by the mean of periodic DFT calculations. These latter were performed using the CRYSTAL17[18] code.

Two different global hybrids (namely PBE0[6,7] and B3LYP[17]) in conjunction with Grimme's dispersion in the Becke-Johnson model (D3BJ)[19-21] were used. A 6-21G (d) and a 5-31G(d) basis sets were applied for C and H atoms, respectively, while a modified m-6-311G(d) basis was used for N atoms.

All three polymorphs can be thermally populated under experimental conditions as evident from computed formation energies reported in Table S4. Both B3LYP and PBE0 predict polymorph 3 (Pol_3) as the most stable with polymorphs 1 and 2 being relatively close in energy.

Table S4. Space group, lattice parameters, formation and cohesive energies computed for TPE-BPAN.

Functional	System	Space Group	a (Å)	b (Å)	c (Å)	α (°)	β (°)	γ (°)	E_{form} (kcal mol^{-1})	E_{coh} (kcal mol^{-1})
B3LYP-D3	Pol_1	P212121	5.132	16.383	38.448	90.000	90.000	90.000	-96.7	386.9
	Pol_2	P212121	16.458	4.844	41.451	90.000	90.000	90.000	-96.0	384.1
	Pol_ 3	P21/C	5.436	40.982	15.377	90.000	106.951	90.000	-98.8	395.2
PBE0-D3	Pol_ 1	P212121	5.183	16.383	38.527	90.000	90.000	90.000	-84.9	339.8
	Pol_ 2	P212121	15.956	5.084	42.243	90.000	90.000	90.000	-85.5	342.1
	Pol_3	P21/C	5.449	41.365	15.380	90.000	106.803	90.000	-88.2	353.0

The computed XRD patterns for these polymorphs are reported below in comparison with the experimental ones.

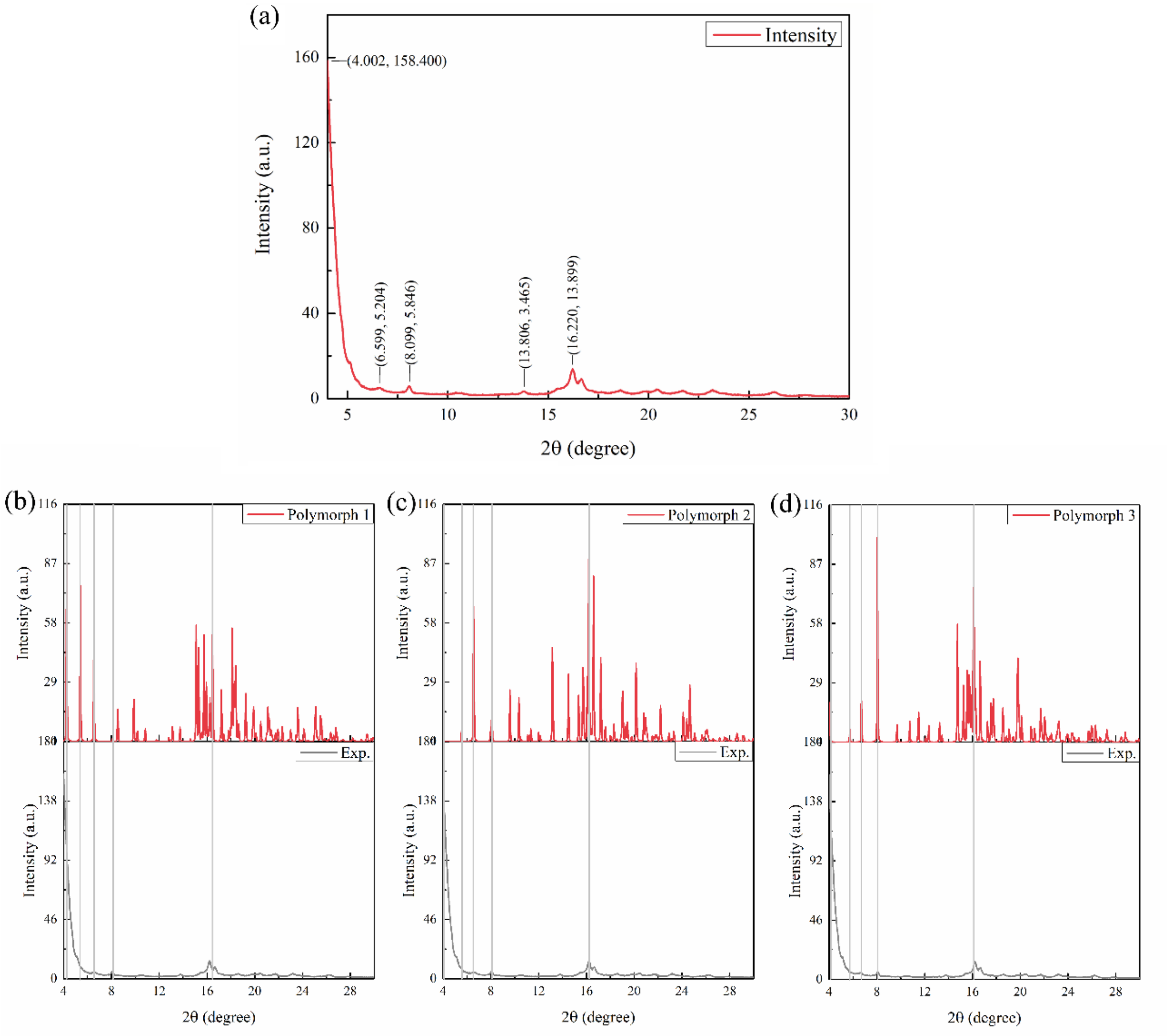

Figure S38. XRD pattern of TPE-BPAN: (a) experimental, (b)-(d) computed at B3LYP

To simulate optical spectra (absorption and emission) in the different polymorphs, the Ewald embedding procedure was applied..[22–26] The effect of the crystalline periodic environment is introduced by the means of a finite charge array located at the atomic position fitted to simulate the exact Magdelung potential in the region of interest. To this end, three zones are defined: a central one containing the molecular unit of interest for optical properties; a second one where charges are kept fixed at the values corresponding to the Mulliken[27] charges computed from periodic DFT calculations; and a third one containing charges fitted to obtain the exact periodic electrostatic potential in zone 1.

In the case of TPE-BPAN, the central zone is constituted by a single molecular unit since direct interaction leading to the formation of excimers can be ignored since no significant π stacking can be observed in the case of all polymers analysed. Optical spectra (absorption and emission) were then computed on the TPE-BPAN molecule embedded in the so obtained charge array using Gaussian16.

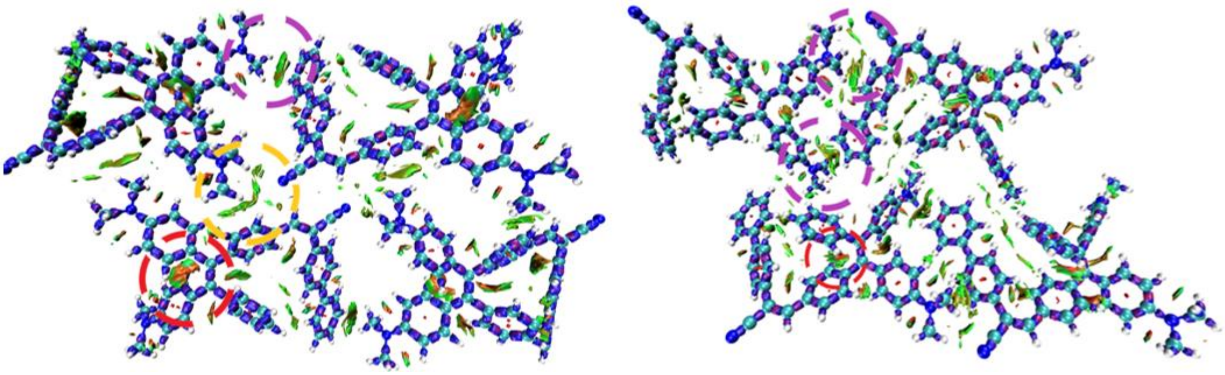

Figure S39: Interaction Region Indicator analysis computed for TPE_BPAN in the Pol_2 and Pol_3 phases. Refer to text for further explanation.

Table S5. Difference in relaxation between solution and aggregate phase (A in degrees) computed at the PBE0 level for the two most stable polymorphs of TPE-BPAN for selected torsional angles. A is defined as A is defined as : $A = |\Delta D_{Ag}| - |\Delta D_{Sol}|$ where $|\Delta D|$ represents the variation (in degree) between the dihedrals computed at the ground and the excited state in solution (Sol) or in the polymorph considered (Ag). Positive A values indicate a larger relaxation in the solid phase while negative values a larger relaxation in solution. The labelling scheme used for the dihedrals is reported in the figure below the table.

ΔD/System	A_{Pol_2}	$A_{Pol\_}$
Δα (°)	-6.093	-1.338
Δβ (°)	-5.62	-3.33
Δγ (°)	-1.199	-10.564
Δζ (°)	-5.78	-4.928
Δφ (°)	5.807	-14.003
Δλ (°)	-1.71	16.358
Δσ (°)	27.804	6.644
Δω (°)	-3.184	-5.39

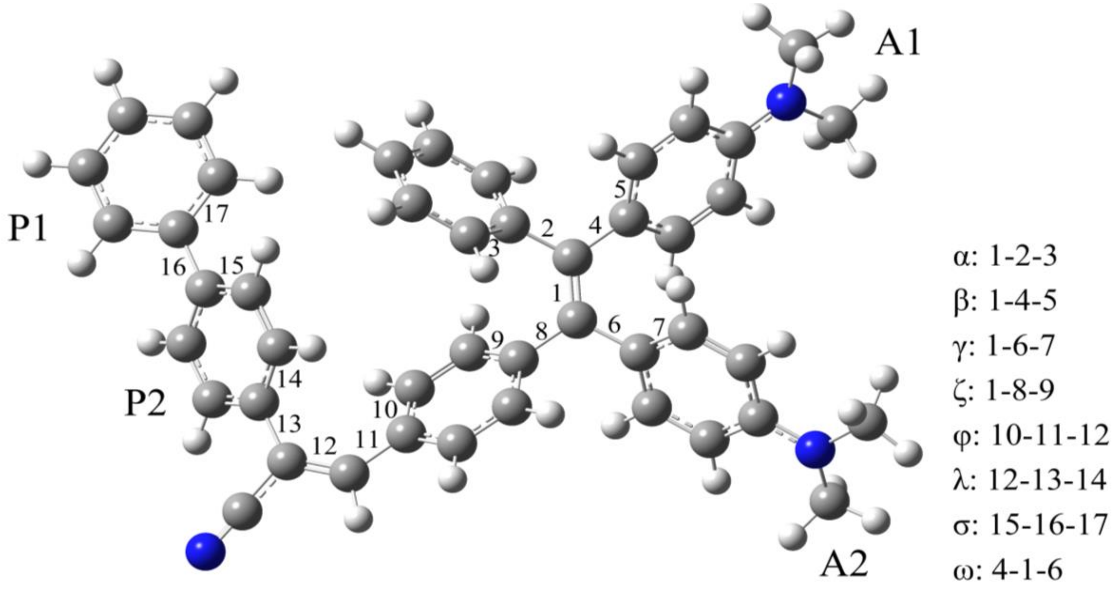

4. References

1. Mori, R.; Iasilli, G.; Lessi, M.; Muñoz-García, A.B.; Pavone, M.; Bellina, F.; Pucci, A. Luminescent Solar Concentrators Based on PMMA Films Obtained from a Red-Emitting ATRP Initiator. *Polym. Chem.* **2018**, *9*, 1168–1177, doi:10.1039/c7py01933e.
2. Wang, E.; Zhao, E.; Hong, Y.; Lam, J.W.Y.; Tang, B.Z. A Highly Selective AIE Fluorogen for Lipid Droplet Imaging in Live Cells and Green Algae. *J. Mater. Chem. B* **2014**, *2*, 2013–2019, doi:10.1039/C3TB21675F.
3. Carlotti, M.; Ruggeri, G.; Bellina, F.; Pucci, A. Enhancing Optical Efficiency of Thin-Film Luminescent Solar Concentrators by Combining Energy Transfer and Stacked Design. *J. Lumin.* **2016**, *171*, 215–220, doi:10.1016/j.jlumin.2015.11.010.
4. Frisch, M. J. T., G. W.; Schlegel, H. B.; Scuseria, G. E.; Robb, M. A.; Cheeseman, J. R.; Scalmani, G.; Barone, V.; Petersson, G. A.; Nakatsuji, H.; Li, X.; Caricato, M.; Marenich, A. V.; Bloino, J.; Janesko, B. G.; Gomperts, R.; Mennucci, B.; Hratchian, H. P.; Ortiz, J. V.; Izmaylov, A. F.; Sonnenberg, J. L.; Williams; Ding, F.; Lipparini, F.; Egidi, F.; Goings, J.; Peng, B.; Petrone, A.; Henderson, T.; Ranasinghe, D.; Zakrzewski, V. G.; Gao, J.; Rega, N.; Zheng, G.; Liang, W.; Hada, M.; Ehara, M.; Toyota, K.; Fukuda, R.; Hasegawa, J.; Ishida, M.; Nakajima, T.; Honda, Y.; Kitao, O.; Nakai, H.; Vreven, T.; Throssell, K.; Montgomery, J. A., Jr.; Peralta, J. E.; Ogliaro, F.; Bearpark, M. J.; Heyd, J. J.; Brothers, E. N.; Kudin, K. N.; Staroverov, V. N.; Keith, T. A.; Kobayashi, R.; Normand, J.; Raghavachari, K.; Rendell, A. P.; Burant, J. C.; Iyengar, S. S.; Tomasi, J.; Cossi, M.; Millam, J. M.; Klene, M.; Adamo, C.; Cammi, R.; Ochterski, J. W.; Martin, R. L.; Morokuma, K.; Farkas, O.; Foresman, J. B.; Fox, D. J., Gaussian 16, Wallingford, CT. **2016**.
5. a) Tomasi, J.; Mennucci, B.; Cammi, R., Quantum Mechanical Continuum Solvation Models. *Chemical Reviews* **2005,** *105* (8), 2999-3094. b) J. Tomasi; B. Mennucci; E. Cancès. “The IEF Version of the PCM Solvation Method: An Overview of a New Method Addressed to Study Molecular Solutes at the QM Ab Initio Level”; *J. Mol. Struct. THEOCHEM,* **1999**, *464* (1–3),

211–226.

6. Adamo, C.; Barone, V., Toward reliable density functional methods without adjustable parameters: The PBE0 model. *The Journal of Chemical Physics* **1999,** *110* (13), 6158-6170.

7. Ernzerhof, M.; Scuseria, G. E., Assessment of the Perdew–Burke–Ernzerhof exchange-correlation functional. *The Journal of Chemical Physics* **1999,** *110* (11), 5029-5036.

8. Krishnan R.; Binkley, J. S.; Seeger, R.; Pople, J. A. Selfconsistent Molecular Orbital Methods. XX. A Basis Set for Correlated Wave Functions; *Cit. J. Chem. Phys*, **1980**, *72*, 650.

9. Huang, K.; Rhys. A. Theory of Light Absorption and Non-Radiative Transitions in F-Centres. **2000**, 74–92.

10. Yanai, T.; Tew, D. P.; Handy, N. C. A New Hybrid Exchange–Correlation Functional Using the Coulomb-Attenuating Method (CAM-B3LYP); *Chem. Phys. Lett.*, **2004**, *393* (1–3), 51–57.

11. Cammi, R., Corni, S. Mennucci, B. Tomasi, J J. Chem. Phys. **2005**, 122, 104513,

12. Caricato, M. Mennucci, B. Tomasi, J. Ingrosso, F., Cammi, R., Corni S., Scalmani, G. J. Chem. Phys., **2006**, 124, 124520

13. Guido, C. A., Jacquemin, D., Adamo, C. Mennucci, B. J. Chem. Theory Comput., **2015**, 11, 5782

14. BIOVIA Materials Studio, Version 2018; Dassault Systèmes, San Diego, CA. **2020**.

15. Mayo, S. L.; Olafson, B. D.; DREIDING: A Generic Force Field for Molecular Simulations; *J. Phys. Chem*, **1990**, *94*, 91101.

16. Cox, S. R.; Williams, D. E.. Representation of the Molecular Electrostatic Potential by a Net Atomic Charge Model; *J. Comput. Chem.*, **1981**, *2* (3), 304–323.

17. Stephens, P.J., Devlin, F.J., Chabalowski, C.F., Frisch, M.J. Phys.Chem. 98 (1994) 11623-11627

18. Dovesi, R.; Erba, A.; Orlando, R.; Zicovich-Wilson, C. M.; Civalleri, B.; Maschio, L.; Rérat, M.; Casassa, S.; Baima, J.; Salustro, S.; Kirtman, B., Quantum-mechanical condensed matter simulations with CRYSTAL. *WIREs Computational Molecular Science* **2018,** *8* (4), e1360.

19. Grimme, S.; Antony, J.; Ehrlich, S.; Krieg, H. A Consistent and Accurate Ab Initio Parametrization of Density Functional Dispersion Correction (DFT-D) for the 94 Elements H-Pu. *J. Chem. Phys.* **2010**, *132*, 154104, doi:10.1063/1.3382344.

20. Grimme S.; Ehrlich S.; Goerigk L. Effect of the Damping Function in Dispersion Corrected Density Functional Theory; *J. Comput. Chem.*, **2011**, *32* (7), 1456–1465.

21. Johnson, E.R.; Becke, A.D. A Post-Hartree-Fock Model of Intermolecular Interactions: Inclusion of Higher-Order Corrections. *J. Chem. Phys.* **2006**, *124*, 174104, doi:10.1063/1.2190220.

22. Presti, D.; Wilbraham, L.; Targa, C.; Labat, F.; Pedone, A.; Menziani, M.C.; Ciofini, I.; Adamo, C. Understanding Aggregation-Induced Emission in Molecular Crystals: Insights from Theory. *J. Phys. Chem. C* **2017**, *121*, 5747–5752, doi:10.1021/acs.jpcc.7b00488.

23. Wilbraham, L.; Louis, M.; Alberga, D.; Brosseau, A.; Guillot, R.; Ito, F.; Labat, F.; Métivier, R.; Allain, C.; Ciofini, I. Revealing the Origins of Mechanically Induced Fluorescence Changes in Organic Molecular Crystals. *Adv. Mater.* **2018**, *30*, 1800817, doi:10.1002/adma.201800817.

24. Wilbraham, L., Adamo, C., Labat, F., Ciofini, I.. Electrostatic embedding to model the impact of environment on photophysical properties of molecular crystals: a self consistent charge adjustment procedure, *J Chem. Theory Comput.*, **2016**, *12* 3316-3324

25. Klintenberg, M.; Derenzo, S.E.; Weber, M.J. Accurate Crystal Fields for Embedded Cluster Calculations. *Comput. Phys. Commun.* **2000**, *131*, 120–128, doi:10.1016/S0010-

4655(00)00071-0.
26. Derenzo, S.E.; Klintenberg, M.K.; Weber, M.J. Determining Point Charge Arrays That Produce Accurate Ionic Crystal Fields for Atomic Cluster Calculations. *J. Chem. Phys.* **2000**, *112*, 2074, doi:10.1063/1.480776.
27. Mulliken, R.S.. Electronic Population Analysis on LCAO-MO Molecular Wave Functions. II. Overlap Populations, Bond Orders, and Covalent Bond Energies; *J. Chem. Phys.*, **1955**, *23* (10), 1841–1846.
